# The genetics of aerotolerant growth in an alphaproteobacterium with a naturally reduced genome

**DOI:** 10.1128/mbio.01487-23

**Published:** 2023-10-31

**Authors:** Amy L. Enright, Amy B. Banta, Ryan D. Ward, Julio Rivera Vazquez, Magdalena M. Felczak, Michael B. Wolfe, Michaela A. TerAvest, Daniel Amador-Noguez, Jason M. Peters

**Affiliations:** 1 DOE Great Lakes Bioenergy Research Center, University of Wisconsin-Madison, Madison, Wisconsin, USA; 2 Pharmaceutical Sciences Division, School of Pharmacy, University of Wisconsin-Madison, Madison, Wisconsin, USA; 3 Microbiology Doctoral Training Program, University of Wisconsin-Madison, Madison, Wisconsin, USA; 4 Laboratory of Genetics, University of Wisconsin-Madison, Madison, Wisconsin, USA; 5 Department of Bacteriology, University of Wisconsin-Madison, Madison, Wisconsin, USA; 6 Department of Biochemistry and Molecular Biology, Michigan State University, East Lansing, Michigan, USA; 7 Department of Biochemistry, University of Wisconsin-Madison, Madison, Wisconsin, USA; 8 Department of Medical Microbiology and Immunology, University of Wisconsin-Madison, Madison, Wisconsin, USA; 9 Center for Genomic Science Innovation, University of Wisconsin-Madison, Madison, Wisconsin, USA; California Institute of Technology, Pasadena, California, USA; University of Minnesota Twin Cities, St. Paul, Minnesota, USA

**Keywords:** CRISPR-Cas9, Mismatch-CRISPRi, Mobile-CRISPRi, essential genes, DNA repair, oxidative stress, ATP synthase, Rnf complex, comparative genomics, metabolomics, membrane potential, anaerobic respiration, *Zymomonas mobilis*, genome reduction, Alphaproteobacteria

## Abstract

Reduced genome bacteria are genetically simplified systems that facilitate biological study and industrial use. The free-living alphaproteobacterium *Zymomonas mobilis* has a naturally reduced genome containing fewer than 2,000 protein-coding genes. Despite its small genome, *Z. mobilis* thrives in diverse conditions including the presence or absence of atmospheric oxygen. However, insufficient characterization of essential and conditionally essential genes has limited broader adoption of *Z. mobilis* as a model alphaproteobacterium. Here, we use genome-scale CRISPRi-seq (clustered regularly interspaced short palindromic repeats interference sequencing) to systematically identify and characterize *Z. mobilis* genes that are conditionally essential for aerotolerant or anaerobic growth or are generally essential across both conditions. Comparative genomics revealed that the essentiality of most “generally essential” genes was shared between *Z. mobilis* and other Alphaproteobacteria, validating *Z. mobilis* as a reduced genome model. Among conditionally essential genes, we found that the DNA repair gene, *recJ*, was critical only for aerobic growth but reduced the mutation rate under both conditions. Further, we show that genes encoding the F_1_F_O_ ATP synthase and *Rhodobacter* nitrogen fixation (Rnf) respiratory complex are required for the anaerobic growth of *Z. mobilis*. Combining CRISPRi partial knockdowns with metabolomics and membrane potential measurements, we determined that the ATP synthase generates membrane potential that is consumed by Rnf to power downstream processes. Rnf knockdown strains accumulated isoprenoid biosynthesis intermediates, suggesting a key role for Rnf in powering essential biosynthetic reactions. Our work establishes *Z. mobilis* as a streamlined model for alphaproteobacterial genetics, has broad implications in bacterial energy coupling, and informs *Z. mobilis* genome manipulation for optimized production of valuable isoprenoid-based bioproducts.

**IMPORTANCE:**

The inherent complexity of biological systems is a major barrier to our understanding of cellular physiology. Bacteria with markedly fewer genes than their close relatives, or reduced genome bacteria, are promising biological models with less complexity. Reduced genome bacteria can also have superior properties for industrial use, provided the reduction does not overly restrict strain robustness. Naturally reduced genome bacteria, such as the alphaproteobacterium *Zymomonas mobilis*, have fewer genes but remain environmentally robust. In this study, we show that *Z. mobilis* is a simplified genetic model for Alphaproteobacteria, a class with important impacts on the environment, human health, and industry. We also identify genes that are only required in the absence of atmospheric oxygen, uncovering players that maintain and utilize the cellular energy state. Our findings have broad implications for the genetics of Alphaproteobacteria and industrial use of *Z. mobilis* to create biofuels and bioproducts.

## INTRODUCTION

Reduced genome bacteria are powerful models for dissecting biological function and streamlined platforms for industrial production. These bacteria have less of the genetic redundancy that often obscures the functions of genes and pathways, simplifying modeling of cellular processes (1, 2). Bacteria with experimentally reduced genome sizes have shown improved properties for industrial applications such as increased genomic stability (3, 4), faster growth rates (5), improved transformation efficiency (6), and ease of genetic manipulation (7), all of which facilitate introduction and maintenance of engineered pathways. However, experimental genome reduction can also result in substantial growth defects (8, 9) and loss of robustness to environmental conditions (10) if the physiology of the bacterium is not well understood and taken into consideration. An appealing alternative approach would be to utilize bacteria with naturally reduced genomes compared with related species. Such bacteria would effectively be “pre-evolved” with the benefits of a reduced genome but with the robustness of environmental strains.

*Zymomonas mobilis* is an emerging model for bacteria with naturally reduced genomes and has excellent properties as an industrial platform. *Z. mobilis* is a member of the highly studied class Alphaproteobacteria but has at least 1,000 fewer genes than closely related species (a total of 1,915 protein-coding genes [11, 12]). This natural genome reduction has created a streamlined metabolism that efficiently ferments sugars to ethanol using the Entner-Doudoroff pathway (13, 14), aiding metabolic modeling efforts (15) and highlighting the promise of *Z. mobilis* as a biofuel producer. Despite its reduced genome, *Z. mobilis* is free living (i.e*.*, not reliant on another organism [16]) and grows quickly to high densities in standard rich medium (e.g., buffered yeast extract and glucose [17]). In contrast, other reduced genome models are often endosymbionts with fastidious growth requirements and dependence on a host (18). *Z. mobilis* growth is robust to diverse environmental conditions including the presence or absence of atmospheric oxygen (aerotolerant anaerobe), high concentrations of ethanol (up to 16% [vol/vol]) (19), and some but not all inhibitors found in plant-derived biofuel fermentation substrates (20). Its safety profile (generally regarded as safe [21]), ease of manipulation in aerobic settings (22), and excellent anaerobic fermentation properties (14) indicate that *Z. mobilis* is both an outstanding model for basic biology of Alphaproteobacteria and a rising industrial workhorse.

Genes required for robust growth of *Z. mobilis* across conditions are understudied, hindering both its use as a reduced genome model and rational engineering efforts to optimize biofuel production. In these contexts, generally and conditionally essential genes are of particular interest because they must be maintained by reduced genomes and are linked to core cellular processes such as carbon metabolism that impact biofuel production (15). Moreover, reduced genomes are thought to harbor a larger fraction of essential genes than larger genomes (18), underscoring the importance of such genes in bacteria with less genetic redundancy. Although transposon (Tn) mutagenesis is often used at the genome scale to identify essential genes (23, 24), previous attempts to apply this approach to *Z. mobilis* were unsuccessful (25), possibly due to the polyploid nature of the *Z. mobilis* chromosome (26–28). Regardless, Tn or other gene disruption approaches alone cannot be used to phenotype genes in the condition where they are essential, since this results in cell death.

With the goal of defining and characterizing essential genes, we previously developed a CRISPRi (clustered regularly interspaced short palindromic repeats interference) gene knockdown system for *Z. mobilis* (29). CRISPRi targets genes for knockdown using a single guide (sg)RNA which directs a catalytically dead (d)Cas9 nuclease to a complementary gene target where the sgRNA-dCas9 complex binds and blocks transcription (30). Our *Z. mobilis* CRISPRi system has several important advantages: it is isopropyl β-D-1-thiogalactopyranoside (IPTG) inducible, titratable with subsaturating inducer or using mismatched sgRNAs (31), and stably integrated into the chromosome without the need for selection and knocks down multi-copy genes simultaneously—features which enable interrogation of both non-essential and essential genes in this plausibly polyploid bacterium.

Here, we combine comparative and functional genomics approaches to establish *Z. mobilis* as a genetic model for Alphaproteobacteria. We use genome-scale CRISPRi to identify genes that are generally or conditionally essential depending on the presence of environmental oxygen. We find that generally essential *Z. mobilis* genes represent a core set of genes that are highly conserved across Alphaproteobacteria. Our sets of *Z. mobilis* aerobic and anaerobic essential genes contain several surprising players, revealing an oxygen-dependent requirement for the RecJ DNA repair protein and critical roles for the ATP synthase and *Rhodobacter* nitrogen fixation (Rnf) complex in the maintenance and utilization of the ion-motive force (IMF) during anaerobic growth. Our studies provide a genetic window into how a naturally reduced genome can be both streamlined and robust.

## RESULTS

### *Z. mobilis* is an emerging model for small-genome, free-living microorganisms

**Fig 1:**
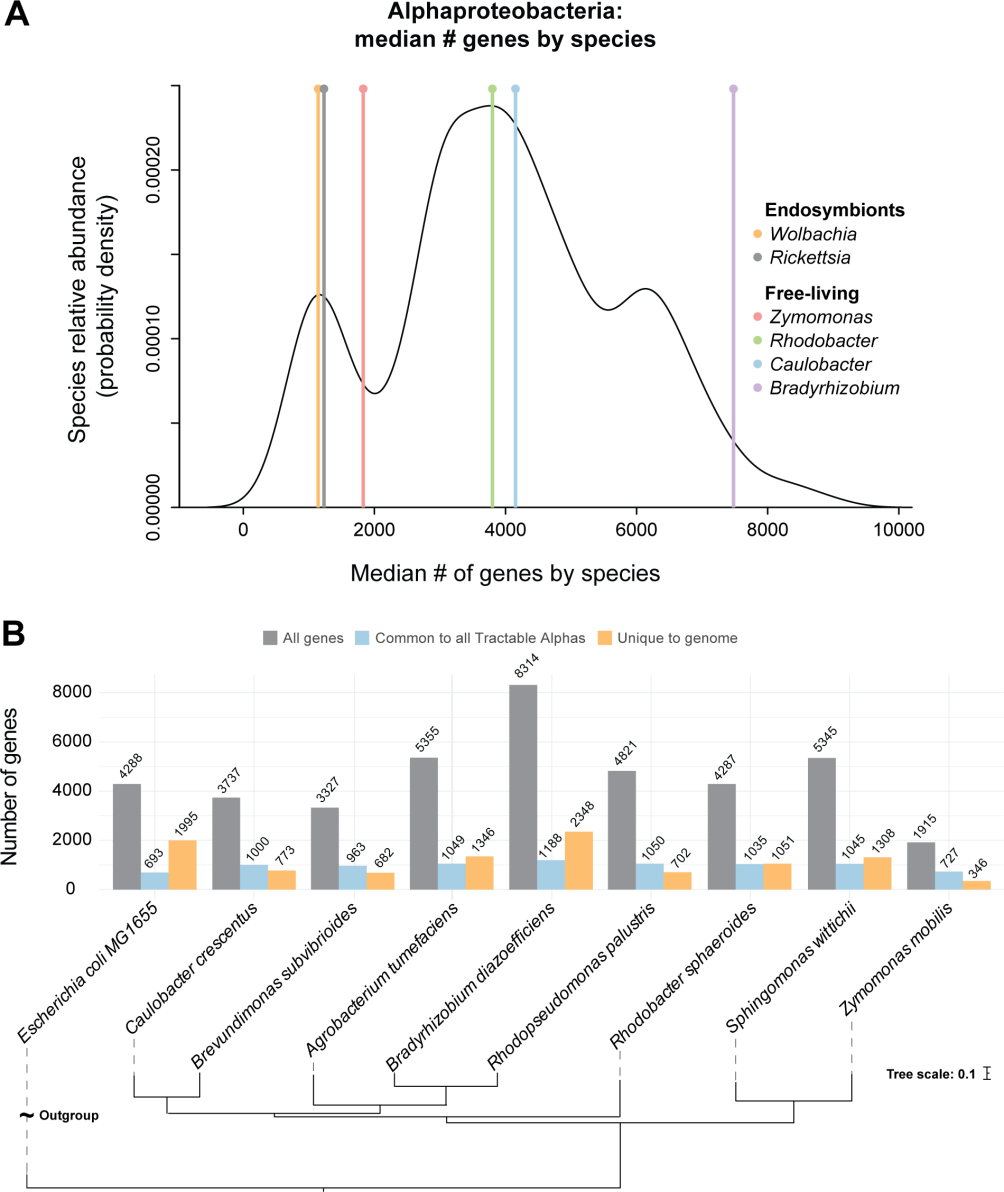
*Zymomonas mobilis* as a small-genome model for Alphaproteobacteria. (**A**) Density plot displaying median number of genes by species of Alphaproteobacteria (black line). Vertical lines mark median number of genes per genus noted. (**B**) Gray lefthand bars, number of genes per organism; blue middle bars, number of genes per organism with homologs in all seven Tractable Alphas (*Caulobacter crescentus*, *Brevundimonas subvibrioides*, *Agrobacterium tumefaciens*, *Bradyrhizobium diazoefficiens*, *Rhodopseudomonas palustris*, *Rhodobacter sphaeroides*, and *Sphingomonas wittichii*). Note these numbers encompass homologous genes within each organism and thus vary slightly across the Tractable Alphas. Orange righthand bars, number of genes per organism with no homologs in any of the seven Tractable Alphas. Phylogenetic tree generated with OrthoFinder using annotated protein-coding genes.

A defining feature of *Z. mobilis* is its naturally small genome of only 1,915 protein-coding genes, which are encoded across one ~2.06-Mb chromosome and four endogenous plasmids (0.03–0.04 Mb each) (12). In contrast, the vast majority of free-living Alphaproteobacteria carry many additional genes (Fig. 1A), including some of the closest *Z. mobilis* relatives, such as *Sphingomonas wittichii* and *Rhodobacter sphaeroides* (~5,300 and ~4,200 genes, respectively) (Fig. 1B) (32, 33). Endosymbiont Alphaproteobacteria (e.g., *Rickettsia* and *Wolbachia*) (Fig. 1A) have even fewer genes than *Z. mobilis* but rely on their host to complement various missing gene functions (34, 35).

To evaluate *Z. mobilis* as a model alphaproteobacterium, we compared the *Z. mobilis* strain ZM4 genome against seven genetically tractable Alphaproteobacteria (“Tractable Alphas”) chosen based on the availability of both a genome sequence and an essential gene list curated from Tn screens (36–41). Given the low genetic diversity within the genus *Zymomonas* (17), ZM4 serves as a strong representation of the genus overall. We found that of the 890 protein-coding genes that are conserved across all seven Tractable Alphas, *Z. mobilis* has orthologs for 701 (~79%) (Fig. S1; Table S2). As expected, this group of genes was enriched for core cellular processes such as translation (false discovery rate [FDR] = 2.9e^−7^) and central metabolic processes (FDR = 9.1e^−6^). *Z. mobilis* also carries relatively few unique genes that do not share orthologs in any of the Tractable Alphas (346 unique genes, fewer than any of the Tractable Alphas) (Fig. 1B); these genes largely encode horizontally transferred elements (e.g., CRISPR-associated immune systems) and hypothetical proteins (Table S2). Genes carried by other Alphaproteobacteria but not by *Z. mobilis* are largely related to oxidative phosphorylation (e.g., type I NADH dehydrogenase and cytochrome c oxidase) and some tricarboxylic acid (TCA) cycle components that are known to be absent in *Z. mobilis* (Table S2) (42–46).

### A whole-genome CRISPRi library for *Z. mobilis*

To interrogate the genetic requirements of this small-genome model species, we constructed a whole-genome knockdown library using our previously developed *Z. mobilis* CRISPRi system (29, 47), a task made further feasible by its minimalistic genome. For nearly all *Z. mobilis* genes (~98%, 1,895/1,933), we successfully designed and cloned four corresponding sgRNAs intended to provide strong knockdown (i.e., the sgRNA sequence is fully complementary to the gene of interest). For the remaining 38 genes, the library contains 1–3 sgRNAs per gene. When assessing gene-level fitness, we considered the behavior of the median sgRNA, a strategy which aims to neutralize any biases incurred from ineffective, off-target, or “bad seed” sgRNAs (48). The library also contains 1,000 non-targeting sgRNAs that act as internal controls.

CRISPRi is inherently polar, where knockdown of an upstream gene within a transcription unit (TU) causes concurrent knockdown of the downstream gene(s) (30, 49). Conversely, “reverse polarity” is a poorly characterized CRISPRi phenomenon in which knockdown of a downstream gene causes decreased expression of the upstream gene(s) (50, 51). Previous studies have observed reverse polarity in *Bacillus subtilis* (49) and to a lesser extent in *Escherichia coli* (48). In contrast, reverse polarity was largely undetected in *Mycobacterium tuberculosis* (52). To investigate if reverse polarity occurs in *Z. mobilis*, we designed sgRNAs targeted along a reporter TU encoding two fluorescent proteins. Our findings revealed that reverse polarity is indeed present in *Z. mobilis*, as knockdown of the downstream sfGFP-encoding gene resulted in decreased expression of the upstream mScarlet reporter, with the strongest reverse-polar effects observed when targeting nearest to the 5′ end of the downstream gene (5.4-fold mScarlet knockdown when targeting the 5′ end of sfGFP and 1.8-fold when targeting 3′ end) (Fig. S2). Similar results were observed in *E. coli* (3.1-fold mScarlet knockdown when targeting the 5′ end of sfGFP and no detectable mScarlet knockdown when targeting 3′ end of sfGFP) (Fig. S2). Together, these results underscore the known importance of interpreting CRISPRi results at the level of TUs. To account for this, we note predicted TUs (Table S5) when reporting gene phenotypes.

### Comprehensive gene function interrogation across oxygen diverse environments

**Fig 2:**
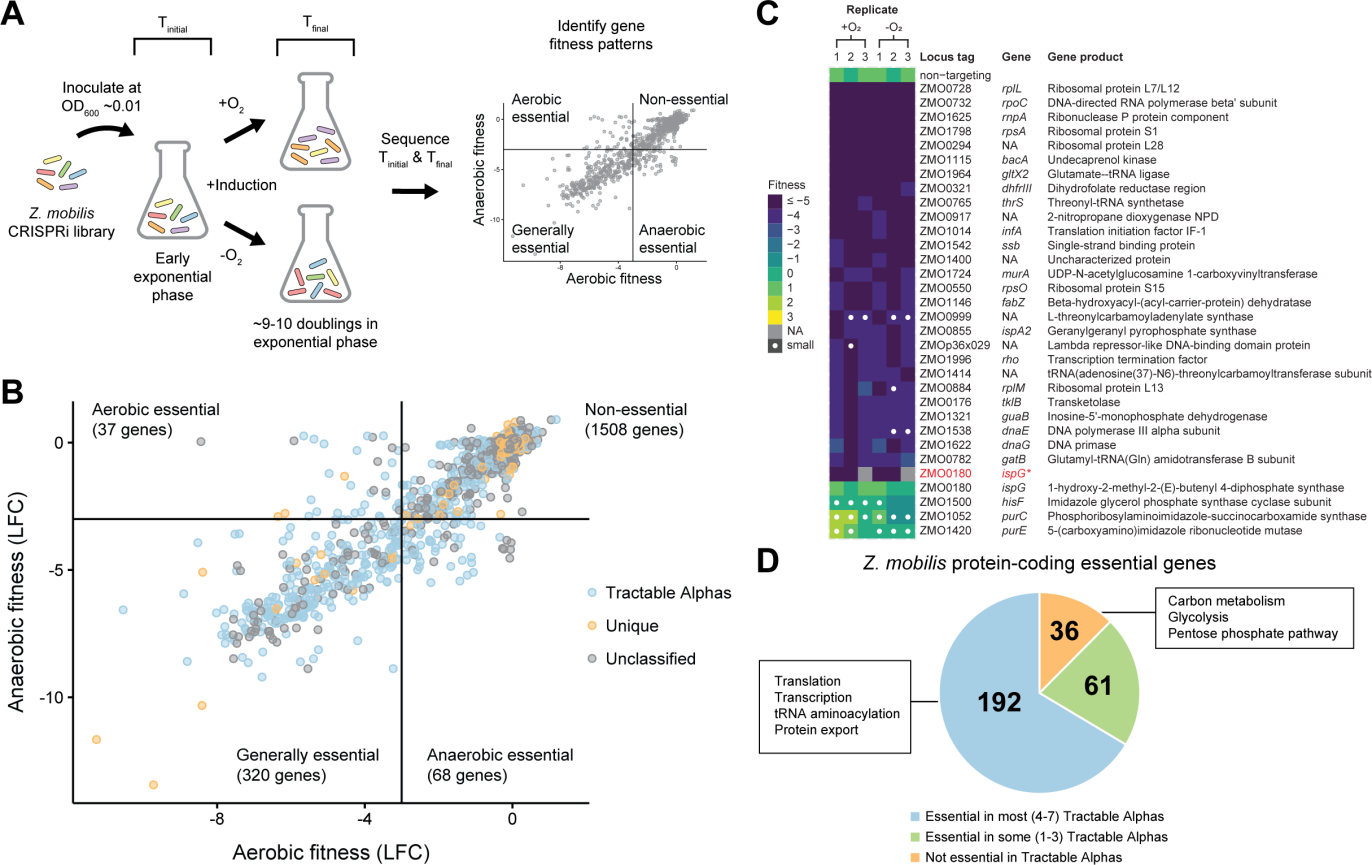
Comprehensive interrogation of gene function and essentiality in *Zymomonas mobilis* using CRISPRi. (**A**) Overview of whole-genome CRISPRi screen to assay *Z. mobilis* gene function during aerobic versus anaerobic growth. (**B**) *Z. mobilis* genes classified as conditionally essential for aerobic versus anaerobic growth, generally essential, or non-essential. Median log_2_ fold change (LFC) for sgRNAs targeting a particular gene for knockdown. Blue, *Z. mobilis* genes with homologs in all seven Tractable Alphas (*C. crescentus*, *B. subvibrioides*, *A. tumefaciens*, *B. diazoefficiens*, *R. palustris*, *R. sphaeroides*, and *S. wittichii*); orange, genes unique to *Z. mobilis* genome, defined by sharing no homologs in any of the seven Tractable Alphas; gray, remaining unclassified *Z. mobilis* genes. (**C**) Validation of generally essential gene phenotypes using a new validation sgRNA that was not included in the CRISPRi library. Heat map displays fitness scores for knockdown mutants assayed individually on spot plates. White dots denote abnormally small colonies (see Materials and Methods). Asterisk marks repeated validation of *ispG* using an sgRNA from our CRISPRi library with an LFC close to the gene median, rather than a new validation sgRNA. Two replicates were assayed for the repeat validation of *ispG*. (**D**) Of the 289 protein-coding *Z. mobilis* essential genes, 192 (~66%) share essential homologs in most (4–7) of the Tractable Alphas, 61 (~21%) share essential homologs in some (1–3) of the Tractable Alphas, and 36 (~12%) share no essential homologs with any of the Tractable Alphas. Gene function enrichments (black boxes) were identified using the STRING database (53).

To understand how *Z. mobilis* employs its small genome to flourish in oxygen diverse environments, we utilized the whole-genome CRISPRi library to identify genes that are conditionally essential for aerobic or anaerobic growth and genes that are generally essential regardless of condition. Library cultures were grown in flasks with saturating concentrations of IPTG (1 mM) to induce CRISPRi and were maintained in the exponential phase for ~10 doublings at which point samples were taken to measure sgRNA spacer abundance by Illumina sequencing (Fig. 2A).

**Fig 3:**
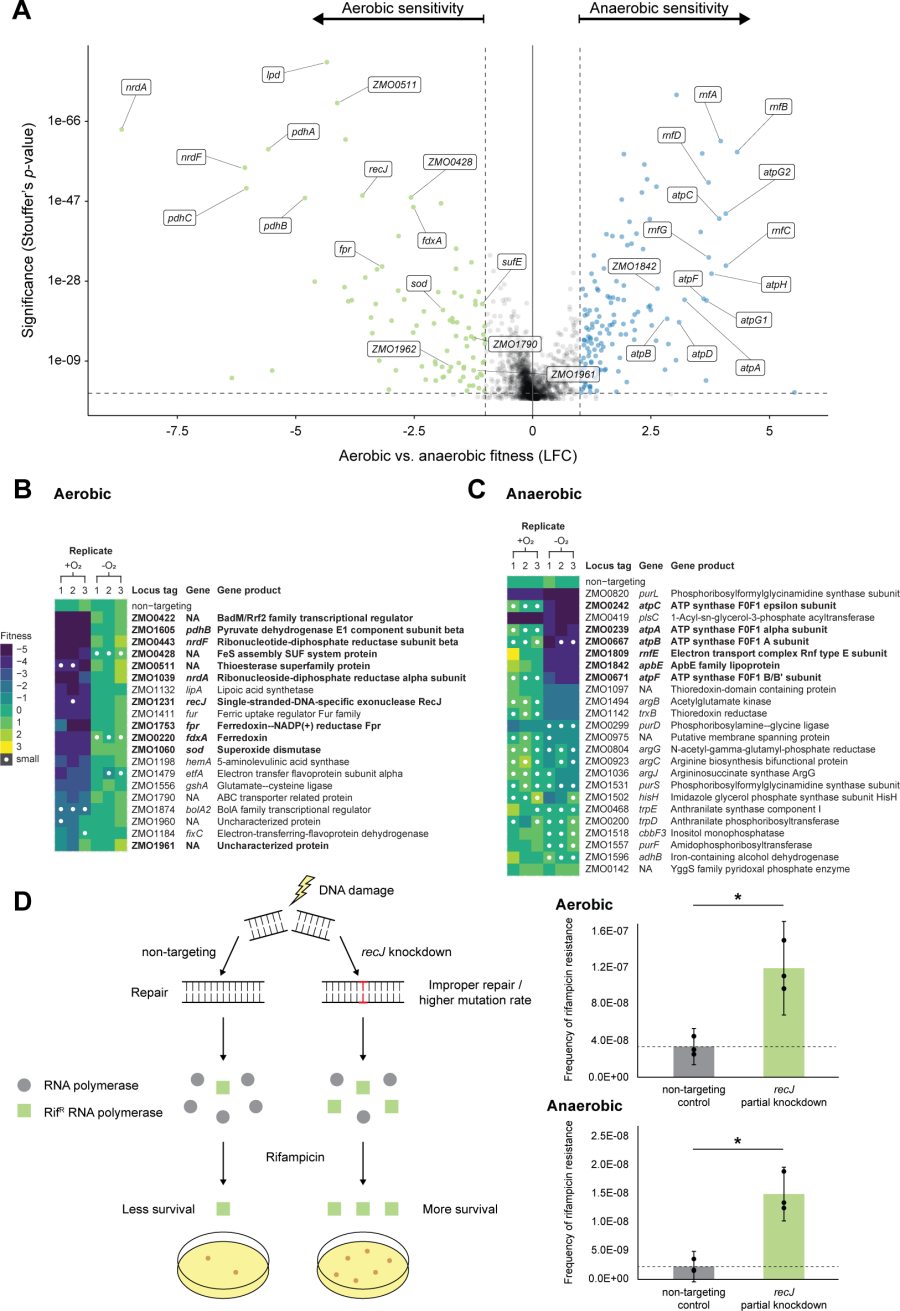
Whole-genome CRISPRi screen reveals condition-specific gene phenotypes for aerobic versus anaerobic growth of *Z. mobilis*. (**A**) Genes with aerobic or anaerobic sensitivity, defined by a fitness score (log_2_ fold change) ≥ |1| and significance (Stouffer’s *P*-value) < 0.05. Dashed lines depict these fitness and significance cutoffs. Green dots, genes with aerobic sensitivity. Blue dots, genes with anaerobic sensitivity. (**B and C**) Validation of (**B**) aerobic-specific sensitivity and (**C**) anaerobic-specific sensitivity gene phenotypes using a new validation sgRNA that was not included in the CRISPRi library. Heat maps display fitness scores for knockdown mutants assayed individually on spot plates. White dots denote abnormally small colonies (see Materials and Methods). (**D**) Left, schematic depicting rifampicin assay as a proxy for mutation rate. In this assay, improper DNA repair elevates the mutation rate and yields increased abundance of surviving rifampicin-resistant (RifR) mutants. Right, frequency of rifampicin resistance for *recJ* partial knockdown versus non-targeting CRISPRi control. Top right, aerobic. Bottom right, anaerobic. Asterisk denotes significance (*P* < 0.01) by two-tailed Student’s *t*-test. Data represent three replicates. Error bars show standard deviation.

Likely due to its small genome size, *Z. mobilis* harbors a larger fraction of essential genes than other model organisms. We identified 320 genes that were essential across both conditions, conservatively defined by log_2_ fold change ≤ −3 and significance ≤ 0.05 (Fig. 2B; Table S6). A similar approach (i.e., LFC < −2) recovered 79% of known *E. coli* essential genes from a genome-scale CRISPRi screen (54), suggesting that our cutoffs largely guard against false positives. Of the 320 generally essential *Z. mobilis* genes, 289 are protein coding; remarkably, these genes represent ~15% of the *Z. mobilis* genome. In comparison, the *E. coli* and *B. subtilis* genomes are made up of only ~7% (307/4,131) and ~6% (257/4,245) protein-coding essential genes, respectively (55–57). Thus, *Z. mobilis* models the unique features of small-genome, free-living species in a way that other model bacteria cannot.

To verify these phenotypes outside of the pooled library context, we chose 31 generally essential genes to assay individually using a new validation sgRNA that was not included in the library. Each knockdown strain was serially diluted and spotted onto plates containing IPTG inducer alongside a non-targeting control and then analyzed following growth for plating and colony size defects (see Materials and Methods). Initially, we successfully recapitulated phenotypes for 27 of the 31 essential gene knockdowns tested (Fig. 2C; Fig. S3). Non-recapitulated phenotypes may result from false positives, specificity to the pooled context, differences in essentiality phenotype during growth on a plate versus in liquid culture, or ineffective validation sgRNAs. For example, we were at first unable to validate the generally essential gene *ispG* (ZMO0180) using the new validation sgRNA. However, when we instead used an sgRNA from our library with an LFC close to the gene median, we successfully validated the *ispG* phenotype outside of the pooled context (Fig. 2C; Fig. S3), bringing the total recapitulated phenotypes to 28 of 31 (~90%).

Comparison of the gene essentiality profile of *Z. mobilis* to the seven Tractable Alphas further solidified its value as a model, small-genome alphaproteobacterium. We found that ~88% of the 289 generally essential *Z. mobilis* genes have homologs that are also essential in at least one other alphaproteobacterium, with ~66% sharing essential homologs in most (≥4) Tractable Alphas (Fig. 2D; Table S2), thus demonstrating substantial agreement between core essential genes of *Z. mobilis* and other Alphaproteobacteria. As expected, essential genes conserved across most Tractable Alphas included fundamental cellular processes such as transcription, translation, and protein export. Genes uniquely essential in *Z. mobilis* were functionally enriched for carbon metabolism (e.g., glycolysis and the pentose phosphate pathway), reflecting a low redundancy, “catabolic highway” (58) ideal for industrial engineering (Fig. 2D; Table S7). Other unique essentials included plasmid-borne, predicted DNA-binding proteins. Given that the native *Z. mobilis* plasmids can be cured without loss of viability (59), we speculate that these proteins are negative regulators of toxic genes that contribute to plasmid maintenance.

### Oxygen-dependent roles for oxygen-resistant enzymes, DNA repair, and ferredoxin/flavodoxin-based redox systems

Tn libraries are often constructed aerobically, obscuring the distinction between genes that are conditionally essential for aerobic growth versus those that are generally essential. In contrast, CRISPRi libraries can be generated aerobically without perturbing genes during the construction process, enabling discovery of genes that are conditionally essential for aerobic growth. Following the same quantitative approach as above, we next identified 37 conditionally essential genes for aerobic growth (LFC ≤ −3 and significance ≤ 0.05 for aerobic growth but not for anaerobic growth) (Fig. 2B and 3A; Table S6). In multiple cases, *Z. mobilis* has redundant enzymes performing similar functions, except one is oxygen tolerant and the other is not. For example, *nrdA* (TU: ZMO1039) and *nrdF* (TU: ZMO0443), which encode class I ribonucleotide reductases (RNRs) that synthesize deoxyribonucleotides for DNA replication and repair, are conditionally essential for aerobic growth, whereas class III RNRs are oxygen sensitive (60). Similarly, genes encoding the pyruvate dehydrogenase complex, which converts pyruvate to acetyl-CoA aerobically or anaerobically (61), including *pdhAB* (TU: ZMO1605-1606; *pdhA, pdhB*), *pdh*C, and *lpd* (TU: ZMO0510-ZMO0513; *pdhC*, ZMO0511, *lpd*, and ZMO0513), are conditionally essential for aerobic growth because the other enzyme responsible for this function (pyruvate formate lyase) is oxygen sensitive (62). The presence of redundant aerobic essentials in an otherwise streamlined genome points to a key role for aerotolerant growth in the natural environment of *Z. mobilis*.

Consistent with previous work that identified *Z. mobilis* genes with increased expression following a shift from anaerobic to oxygen-replete conditions (22), knockdown of known oxidative stress genes such as *sod* (superoxide dismutase; TU: ZMO1060) and iron-sulfur cluster assembly (*suf*) genes including *sufE* (TU: ZMO1067-1068; *sufE*, ZMO1068), ZMO0422 (homolog of transcriptional regulator IscR), and ZMO0428 (TU: ZMO0422-0429; ZMO0422, *sufB*, *sufC*, *sufD*, *sufS*, ZMO0428, and ZMO0429) also showed oxygen-specific fitness defects (Fig. 3A and B; Table S6).

Interestingly, the DNA repair gene *recJ* (TU: ZMO1231) was also found to be conditionally essential for aerobic growth, and this phenotype was validated outside the pooled context (Fig. 3A and B). Although non-essential in *E. coli* (63), previous work has shown that *recJ* is essential across the Alphaproteobacteria (64) and in *Deinococcus radiodurans* (65, 66) for aerobic growth. However, these studies did not (or could not, in the case of strict aerobes) compare the *recJ* phenotype during aerobic versus anaerobic growth. *recJ* knockdowns in *Z. mobilis* showed no apparent growth phenotype under anaerobic conditions. To understand the role of RecJ in alphaproteobacterial physiology, we examined the mutation frequency in *recJ* knockdown strains. We found that RecJ suppresses the mutation frequency in both aerobic and anaerobic conditions. Because full knockdown of *recJ* is lethal aerobically, we used a subsaturating concentration of inducer to create partial knockdowns. To calculate mutation frequency, we measured the number of rifampicin-resistant colony forming units (CFU) in non-targeting and *recJ* partial knockdown strains. Knockdown of *recJ* increased the mutation frequency in both aerobic (3.6-fold) and anaerobic conditions (6.8-fold; Fig. 3D); thus, we establish a role for RecJ in mutation suppression in *Z. mobilis* that may be applicable to other Alphaproteobacteria. However, the mechanistic rationale for *recJ* essentiality during aerobic growth remains elusive and requires further studies. We were additionally able to associate an oxygen-specific phenotype with unannotated or poorly annotated genes including the TU encoding ZMO1961-ZMO1962 (uncharacterized protein and putative histidine kinase, respectively) and ZMO1790 (TU: ZMO1790; predicted heme-related transporter [67]) (Fig. 3A and B; Table S6). We report these and other uncharacterized genes with phenotypes from our screen in Table S7. Finally, we identified ferredoxin/flavodoxin (Fd/Fld)-based redox systems specifically required for aerobic growth including *fdxA* (TU: ZMO1753) and *fpr* (Fd/Fld reductase; TU: ZMO0220). As we will highlight in the next section, such redox functions play a similarly indispensable role in anaerobic growth, though the relevant genetic players may differ by condition.

### Rnf respiratory complex and F_1_F_O_ ATP synthase are essential for anaerobic growth

We next examined the 68 genes that were conditionally essential for growth in the absence of oxygen (LFC ≤ −3 and significance ≤ 0.05 for anaerobic growth but not for aerobic growth) (Table S6). Strikingly, TUs encoding either the Rnf complex (TU: ZMO1808-1814; *rnfH*, *rnfE*, *rnfG*, *rnfD*, *rnfC*, *rnfB*, and *rnfA*) or the F_1_F_O_ ATP synthase (TU-1: ZMO0238-0241; *atpH*, *atpA*, *atpG1*, and *atpD*). TU-2: ZMO0242; *atpC*. TU-3: ZMO0669-0671; *atpG2*, *atpF*) were conditionally essential for anaerobic growth. Of note, while *rnf* knockdown was inconsequential for growth in the presence of oxygen (median aerobic LFC for *rnf* genes = −0.2), *atp* knockdown resulted in a mild aerobic fitness defect (median aerobic LFC for *atp* genes = −1.2) (Table S6).

The F_1_F_O_ ATP synthase reversibly couples ion transport with the synthesis or hydrolysis of ATP. Rnf, originally named for its role in *Rhodobacter* nitrogen fixation, is similarly reversible and couples ion transport with electron transfer between ferredoxin and pyridine nucleotides (68). A previous Tn study by Deutschbauer et al. also observed that *rnf* genes are required for anaerobic growth of *Z. mobilis*; however, the mechanism underlying this phenotype was not explored further (67). The same group proposed ZMO1842 to be *rnfF* (TU: ZMO1842-1842; possibly *rnfF*, *rseC*) based on cofitness with other *rnf* genes. This gene was also conditionally essential for anaerobic growth in our screen (Table S6), but it presently is not annotated as *rnfF* and thus was not included in our subsequent *rnf* analyses.

**Fig 4:**
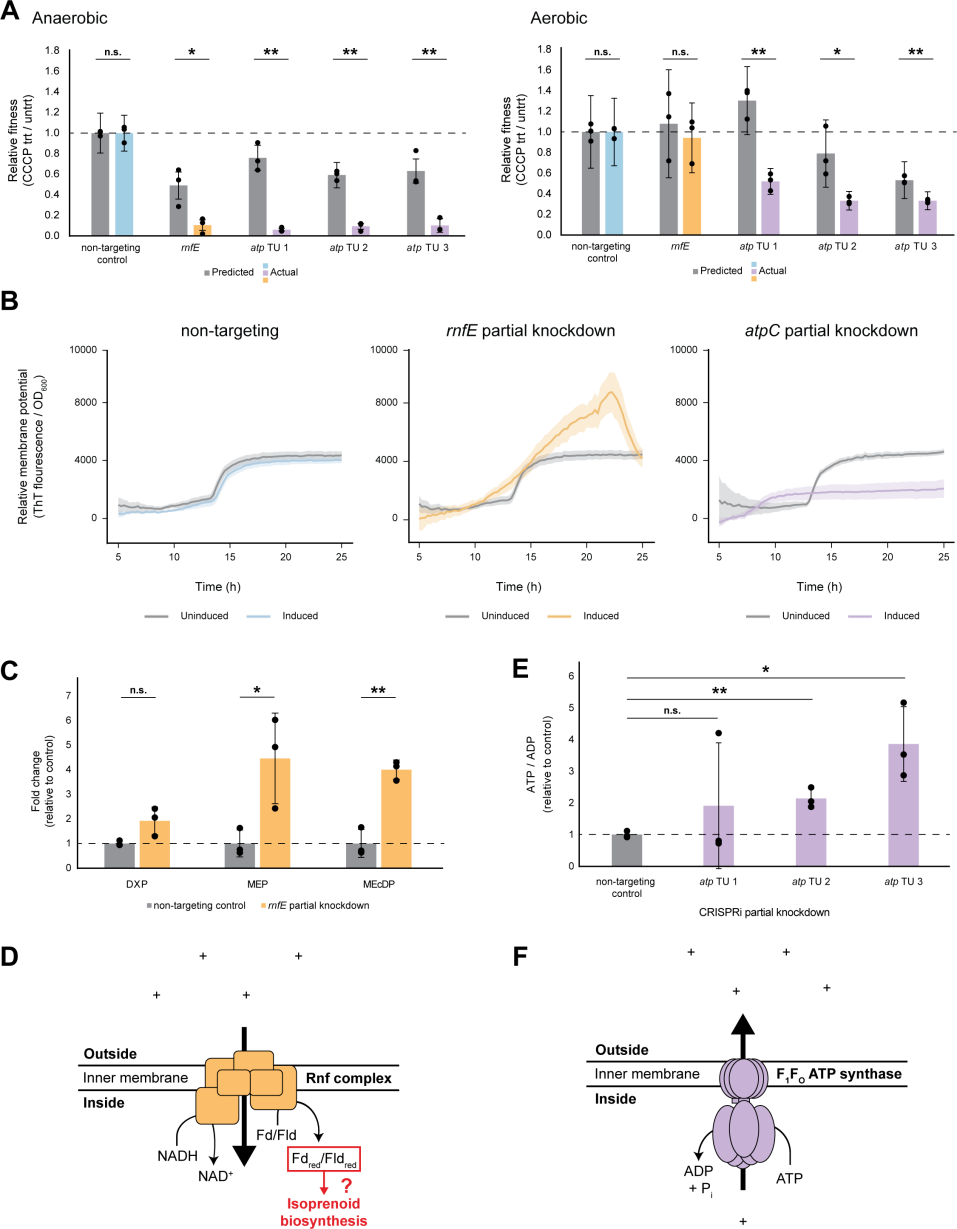
Functional analysis of the *Z. mobilis* F_1_F_O_ ATP synthase and Rnf complex during anaerobic growth. n.s., not significant; *P < 0.05 and **P < 0.01 by two-tailed Student’s *t*-test. Error bars show standard deviation. TU, transcription unit. *atp* TU 1 knockdown targets *atpA*; *atp* TU 2 knockdown targets *atpC*; *atp* TU 3 knockdown targets *atpF*. (**A**) Fitness of *rnf* or *atp* gene knockdowns during disruption of the electrochemical gradient by sublethal challenge with the protonophore CCCP. Fitness values are relative to the non-targeting control (horizontal dashed line). Gray lefthand bars represent predicted fitness by the multiplicative model (fitness in CCCP × fitness of partial knockdown). Colored righthand bars show measured fitness of non-targeting control (blue), *atp* knockdowns (purple), or *rnf* knockdown (orange). Left, anaerobic; right, aerobic. Data represent three experiments with two biological replicates each. (**B**) Thioflavin T (ThT) assay to measure relative membrane potential of (left) non-targeting CRISPRi control, (middle) *rnfE* partial knockdown, or (right) *atpC* partial knockdown. Gray lines, uninduced; colored lines, induced partial knockdown. Shaded ribbons represent one standard deviation from the mean. Data represent six replicates. (**C**) Mass spectroscopy metabolomics measurement of methylerythritol 4-phosphate (MEP) pathway intermediates for (lefthand gray bars and dashed line) non-targeting CRISPRi control and (righthand orange bars) *rnfE* partial knockdown. DXP, 1-deoxy-D-xylulose 5-phosphate; MEP, 2-C-methyl-D-erythritol 4-phosphate; MEcDP, 2-C-methyl-D-erythritol-2,4-cyclodiphosphate. Data represent three replicates. (**D**) Speculative model for Rnf function during *Z. mobilis* anaerobic growth: Rnf utilizes the ion-motive force to reduce ferredoxin/flavodoxin (Fd/Fld), and reduced Fd/Fld (Fd_red_/Fld_red_) powers important biological processes such as isoprenoid biosynthesis via the MEP pathway. (**E**) Mass spectroscopy metabolomics measurement of the ATP/ADP ratio for (gray bars and dashed line) non-targeting CRISPRi control and (purple bars) *atp* partial knockdowns. Data represent three replicates. (**F**) Model for F_1_F_O_ ATP synthase function during *Z. mobilis* anaerobic growth: ATP is hydrolyzed to pump ions across the inner membrane.

We sought to understand the potential mechanism driving the *rnf* and *atp* anaerobic growth phenotypes. First, we tested whether the complexes depend on the electrochemical gradient using carbonyl cyanide 3-chlorophenylhydrazone (CCCP), an ionophore that dissipates the proton gradient. We reasoned that partial knockdown of *rnf* and *atp* genes could act as a sensitized genetic background against which to test CCCP challenge. Given that the *atp* genes are encoded across three operons in *Z. mobilis*, we targeted one gene per *atp* operon for knockdown (*atpA*, *atpC*, and *atpF*). For *rnf* knockdown, *rnfE* was targeted. Partial knockdown was induced with a subsaturating concentration of IPTG inducer with the goal of achieving an intermediate level of fitness, rather than a specific expression level (Fig. S4). We found that, under anaerobic conditions, *rnf* and *atp* knockdowns had increased sensitivity to a normally sub-lethal concentration of CCCP, demonstrating their interdependence on IMF (Fig. 4A). Consistent with our screen results, when the assay was repeated aerobically, the *rnf* knockdown mutant behaved like the non-targeting control while *atp* knockdowns retained some sensitivity to CCCP, though to a lesser extent than was seen in the absence of oxygen (Fig. 4A).

Given that the reactions carried out by both complexes are reversible, multiple models could explain the synergy of the ion gradient with F_1_F_O_ ATP synthase and Rnf. In each case, interdependence with the ion gradient could indicate either that the complex functions to establish the gradient (a producer) or that it depends on the gradient to carry out its essential function (a consumer). Thus, we next investigated the directionality of both complexes during anaerobic growth.

Two lines of evidence support the hypothesis that Rnf operates as a membrane potential consumer during anaerobic growth. First, a ThT membrane potential assay, wherein increased ThT fluorescence indicates stronger membrane potential (69, 70), revealed that partial knockdown of *rnfE* yielded stronger membrane potential than an uninduced control (Fig. 4B; Fig. S5). Second, mass spectrometry metabolomics analysis of the *rnfE* partial knockdown uncovered an accumulation of intermediate metabolites in the methylerythritol 4-phosphate isoprenoid biosynthesis pathway. Iron-sulfur cluster enzymes involved in the MEP pathway require reduced ferredoxin/flavodoxin (Fd_red_/Fld_red_) (Fig. S6) (22). We observed ≥ four-fold accumulation of two intermediates, MEP and MEcDP (Fig. 4C). Thus, we infer that *rnfE* knockdown hinders Fd/Fld reduction, leading to a bottleneck in MEP pathway flux. As such, our data are consistent with the conclusion that, in the absence of oxygen, Rnf uses the ion gradient to power the otherwise unfavorable transfer of electrons from NADH to Fd/Fld, which in turn drives essential processes such as isoprenoid biosynthesis (Fig. 4D).

Conversely, analysis of the F_1_F_O_ ATP synthase agrees with previous evidence suggesting the ATP synthase operates as a membrane potential generator (71–75). Partial knockdown of *atpC* resulted in lower membrane potential than an uninduced control (Fig. 4B). Furthermore, we reasoned that quantifying the ATP/ADP ratio for the *atpC* partial knockdown versus an uninduced control using metabolomics may illuminate the reaction direction. For example, if partial knockdown of *atp* genes results in an accumulation of ATP, this would suggest that, in the absence of knockdown, the F_1_F_O_ ATP synthase is functioning to hydrolyze ATP and pump ions outside the cytoplasm. Indeed, the ATP/ADP ratio increased relative to the non-targeting control for the two *atp* knockdowns with the clearest signal (Fig. 4E). This accumulation of ATP in the *atp* knockdown mutants indicates that during anaerobic growth in rich medium, the F_1_F_O_ ATP synthase hydrolyzes ATP to generate IMF (Fig. 4F).

## DISCUSSION

Alphaproteobacteria are fascinating models for fundamental biological processes as well as microbial powerhouses for industrial production of green energy. Despite this importance, core genes in Alphaproteobacteria are understudied due to the lack of both a simplified model genome and genetic tools capable of phenotyping all genes. This work advances our understanding of alphaproteobacterial genes by establishing *Z. mobilis* as a streamlined, model microbe with a naturally reduced genome and employing a genome-scale, CRISPRi strategy for comprehensive phenotyping. We identified generally and conditionally essential genes that underpin the aerotolerant lifestyle of *Z. mobilis* and found broad conservation of these genes in other Alphaproteobacteria. Our analysis of Rnf and ATP synthase respiratory complexes highlights critical functions in maintaining/consuming IMF and points to a key role of Rnf in isoprenoid synthesis. Both our CRISPRi strategy and resulting insights into core gene functions are readily applicable to Alphaproteobacteria and beyond.

CRISPRi inducibility and titratability are invaluable for identifying conditional essentiality phenotypes. Although Tn-seq remains broadly useful for identifying non-essential gene phenotypes without the need to design guide libraries, CRISPRi has some clear advantages. Because genes are instantly inactivated during Tn library construction, genes that are essential under the condition the library was constructed in are lost from the pool before downstream phenotyping experiments can begin. The only way to mitigate this issue would be to construct a new Tn library for every condition assayed—a considerable and impractical burden for investigators that are interested in probing many conditions to evaluate gene networks. CRISPRi inducibility mitigates this issue by separating library construction from fitness assays. As a result, we were able to define genes essential for aerobic growth despite constructing our CRISPRi library aerobically. An important example from our study is *recJ*, which has been defined as “essential” in previous alphaproteobacterial Tn screens (64), but what we show is conditionally essential during aerobic growth and dispensable anaerobically. Other genes that are canonically associated with oxygen stress (e.g., *sod*) may also appear to be generally essential if Tn libraries are constructed aerobically. Titrating CRISPRi knockdowns allowed us to investigate the phenotypes of conditionally essential genes in the condition for which they are essential. This enabled us to define the mutator phenotype of *recJ* and IMF altering phenotypes of *rnf* and *atp* genes. Given the portability of CRISPRi (e.g., Mobile-CRISPRi [76]), we anticipate its broad utility in characterizing conditional essentiality.

We report that the broadly conserved respiratory complexes, Rnf and ATP synthase, are conditionally essential during *Z. mobilis* anaerobic growth and provide possible causes for their essentiality. Rnf complexes are widespread across prokaryotes and couple ion-motive force (Na^+^ or H^+^) with reversible Fd/Fld:NAD^+^ oxidoreductase activity (that is, oxidation of Fd_red_/Fld_red_ and reduction of NAD^+^ and vice versa) to accomplish diverse biological roles (68). In some anaerobes, Rnf is required for energy conservation: oxidation of Fd_red_/Fld_red_ is coupled with ion transport to establish ion-motive force necessary for ATP generation. Recently, the Rnf-ATP synthase supercomplex from *Thermotoga maritima* was purified and conclusively demonstrated to operate in this manner *in vitro* (77). In other organisms, ion transport occurs in the reverse direction, with ion-motive force powering the transfer of electrons from NADH to Fd/Fld. Canonically, Fd_red_/Fld_red_ then acts as a biologically powerful reductant and donates electrons to nitrogenase, a required step in N_2_ fixation (78, 79). *Z. mobilis* is known to fix N_2_ anaerobically in minimal medium lacking biologically available nitrogen (ammonium) (80). Under these conditions, *rnf* and other *nif* cluster genes associated with N_2_ fixation are upregulated accordingly (81), and their functions may be further regulated by protein phosphorylation status (82).

The present study extends the role of Rnf by demonstrating that Rnf is required for anaerobic growth and MEP pathway flux in *Z. mobilis*, even when ammonium is abundant. Here, using a multi-modal approach (genetic sensitivity to electrochemical gradient disruption, ThT membrane potential assay, and metabolomics), we demonstrate that Rnf is a consumer of the ion gradient (Fig. 4), suggesting that it uses IMF to power reduction of Fd/Fld. Furthermore, we show that *rnfE* knockdown results in accumulation of MEP isoprenoid biosynthesis pathway intermediates (Fig. 4C). We suggest that MEP pathway flux is disrupted due to a deficiency in Fd_red_/Fld_red_, which donate electrons to the iron-sulfur cluster enzymes IspG and IspH in the MEP pathway (22, 83). Thus, this downstream disfunction of the essential MEP pathway may explain the essentiality of Rnf for anaerobic growth, in part or in full. Isoprenoids produced through the MEP pathway are biologically invaluable. In bacteria, isoprenoids act as electron carriers (quinones), pigments (carotenoids), membrane components (hopanoids), and signaling molecules (84). Given that isoprenoid biosynthesis is essential under all conditions for *Z. mobilis* (Table S6 and references) (29, 85, 86), the link between Rnf and MEP pathway flux during anaerobic growth suggests a separate mechanism may exist for generating Fd_red_/Fld_red_ to maintain flux during aerobic growth. Indeed, ferredoxin (*fdxA*; ZMO0220) and Fd/Fld reductase (*fpr*; ZMO1753) were both conditionally essential for aerobic growth in our screen. Given that *fpr* is also one of the most highly upregulated genes following oxygen exposure (22), we speculate that these genes may replenish Fd_red_/Fld_red_ aerobically, while Rnf performs this function anaerobically.

Ongoing engineering efforts aim to increase isoprenoid production by *Z. mobilis* for industrial use as therapeutics, food additives, fragrances, and sustainable biofuels (87). For example, Martien et al. observed that upregulation of *fpr*, *ispG*, and the iron-sulfur cluster assembly operon (*suf*) upon oxygen exposure coincided with improved flux through the MEP pathway, suggesting that manipulation of these genes could yield isoprenoid production strains with high MEP pathway flux (22). This proposal is supported by a work in *E. coli*, where overexpression of Fpr, FldA, and MEP pathway enzymes increased flux through the MEP pathway (88). Furthermore, Khana et al. demonstrate that the iron-sulfur cluster enzymes IspG and IspH can modulate bottlenecks in the MEP pathway, and the authors point to electron-resupplying accessory proteins as possible engineering targets to further increase MEP pathway flux (83). The present work adds Rnf to this toolkit of potential engineering targets for enhancing isoprenoid production by *Z. mobilis*, especially for anaerobic industrial fermentations.

We also find that the F_1_F_O_ ATP synthase is conditionally essential for anaerobic growth. We further demonstrate that the F_1_F_O_ ATP synthase performs the essential function of hydrolyzing ATP to pump ions across the inner membrane and establish IMF (i.e., running in reverse). This role is consistent with previous conclusions surrounding *Z. mobilis* F_1_F_O_ ATP synthase function (71, 73, 74, 88) , including work showing that addition of CCCP affects membrane potential but not intracellular concentration of ATP (75). Our work both confirms this literature and additionally demonstrates that this function is essential for *Z. mobilis* anaerobic growth.

Our screen and CCCP sensitivity data also revealed an interesting contrast between the effects of *rnf* and *atp* knockdown on aerobic fitness. Specifically, while *rnf* knockdown appears to have no detectable impact on aerobic growth, knockdown of *atp* results in a mild fitness defect in the presence of oxygen (Fig. 4A; Table S6). This discrepancy could be explained by the possibly redundant function of *atp* with the *Z. mobilis* aerobic respiratory chain, which has an elusive function but may contribute to ion gradient generation and/or detoxification of oxygen (44, 72).

Given the clear connection between Rnf and F_1_F_O_ ATP synthase in *Thermotoga maritima* (77), it is tempting to speculate an equal-but-opposite model for *Z. mobilis* wherein the F_1_F_O_ ATP synthase establishes an ion gradient that is then directly used by Rnf. It is especially tempting given the long-anticipated discovery of a IMF-dissipating counterpart to F_1_F_O_ ATP synthase function (72, 73). Our data, however, point to additional biological complexity. If the main function of the F_1_F_O_ ATP synthase was to supply IMF for Rnf function, *atp* knockdown should yield a similar accumulation of MEP metabolites as occurs for *rnf* knockdown. However, we did not observe a consistent trend in MEP pathway metabolites for *atp* knockdown strains (Fig. S7). Multiple possible explanations exist. For example, disruption of the ion gradient by *atp* knockdown likely has widespread effects on cellular physiology (e.g*.,* in motility and transport) which may convolute biological outcomes, there may be additional ion translocators contributing to the ion gradient, and there may be other MEP pathway regulatory mechanisms at play (22, 83).

Herein, we establish *Z. mobilis* as a valuable genetic model for Alphaproteobacteria and deepen our understanding of how this naturally reduced genome bacterium adapts to the presence or absence of environmental oxygen. We anticipate that its use as a streamlined model with decreased genetic redundancy will simplify functional analysis of the basic biology of Alphaproteobacteria. We further expect that the novel biology gleaned from this study will aid in the development of prolific *Z. mobilis* strains for biofuel production.

## MATERIALS AND METHODS

### Strains and growth conditions

Strains and media recipes are listed in Table S1. *Escherichia coli* was grown in LB broth, Lennox (BD240230) at 37°C aerobically in a flask with shaking at 250 rpm, in a culture tube on a roller drum, or in a 96-well deep well plate with shaking at 900 rpm. *Zymomonas mobilis* was grown at 30°C aerobically or anaerobically (anaerobic chamber with 5% CO_2_, 5% H_2,_ and balance N_2_) in rich medium glucose (RMG), *Zymomonas* rich defined medium (ZRDM), or *Zymomonas* minimal medium (ZMM) either statically in a culture tube or deep well plate for polarity experiments, in flasks with stirring at 150 rpm for library experiments, or in a Tecan Sunrise microplate reader statically with 60 s shaking at high intensity prior to optical density at 600 nm (OD_600_) reads every 15 min for growth curves. Media were solidified with 1.5%–2% agar for growth on plates. Antibiotics were added when necessary: *E. coli* (100 µg/mL ampicillin (amp), 100 µg/mL carbenicillin (carb), 20 µg/mL chloramphenicol (cm), and *Z. mobilis* (100 µg/mL cm). Diaminopimelic acid (DAP) was added at 300 µM to support growth of *dap^−^ E. coli* strains. Isopropyl β-D-1-thiogalactopyranoside (0.1–1 mM) was added where indicated. Strains were preserved in 15% glycerol at −80°C.

### General molecular biology techniques

Plasmids and oligonucleotides are listed in Table S1. *pir*-dependent plasmids were propagated in *E. coli* strain BW25141 (sJMP146). Plasmids were purified using the GeneJet Plasmid Miniprep Kit (Thermo K0503), the QIAprep Spin Miniprep Kit (Qiagen 27106), or the Purelink HiPure Plasmid Midiprep Kit (Invitrogen K210005). Plasmids were digested with restriction enzymes from NEB and ligated using T4 DNA ligase (NEB M0202). DNA fragments were amplified using Q5 DNA polymerase (NEB 0491). PCR products were purified using the Monarch PCR & DNA Cleanup Kit (NEB T1030). Plasmids were transformed into electrocompetent *E. coli* cells using a Bio-Rad Gene Pulser Xcell using the Ec1 program (0.1-cm cuvette, 1.80 kV, and 1 pulse). Oligonucleotides were synthesized by Integrated DNA Technologies (Coralville, IA) or Agilent (Santa Clara, CA). Sequencing was performed by Functional Biosciences (Madison, WI) or the University of Wisconsin Biotechnology Center Next Generation Sequencing Core (Madison, WI).

### Alphaproteobacterial genome size analysis

Genome size analysis was performed using all complete National Center for Biotechnology Information (NCBI) genome entries for Alphaproteobacteria with a specific genus (i.e*.,* not “*Candidatus*,” “alpha,” or “uncultured”) as of 7 July 2021. The median number of gene coding sequences (CDS) was calculated for each species and each genus.

### Comparative analysis of conserved and essential genes

OrthoFinder (89, 90) was used to identify orthologous groups among organisms (Table S2). Annotated genomes of Alphaproteobacteria associated with published Tn-seq experiments (“Tractable Alphas”) obtained from the NCBI and corresponding published lists of essential genes for these organisms (36–41) were analyzed. The model organism *E. coli* served as a comparator (55, 56). See Table S3 for additional information on digital resources and links to custom scripts.

### *Z. mobilis* Mobile-CRISPRi individual gene and gene library construction

sgRNAs were designed to knock down all genes in *Z. mobilis* ZM4 using a custom Python script and GenBank CP023715.1 as detailed in reference (29). sgRNA-encoding sequences were cloned between the BsaI sites of Mobile-CRISPRi (MCi) plasmid pJMP2480. Methodology for cloning individual sgRNAs was described previously in detail (29, 47). Briefly, two 24-nucleotide (nt) oligonucleotides encoding an sgRNA were designed to overlap such that when annealed, their ends would be complementary to the BsaI-cut ends on the vector.

The pooled CRISPRi library was constructed by amplification of sgRNA-encoding spacer sequences (Table S4) from a custom-pooled oligonucleotide library (SurePrint G7221A, Agilent) followed by ligation into the BsaI-digested MCi plasmid. Specifically, three pools of sgRNA-encoding inserts were generated by PCR amplification with primers oJMP197 and oJMP198 (Z1-genes), oJMP463 and oJMP464 (Z2-controls), and oJMP465 and oJMP466 (Z3-mismatches) from a 78-nt custom-pooled oligonucleotide library with the following conditions per 300 µL reaction: 60 µL Q5 buffer, 9 µL GC enhancer, 6 µL 10 mM each dNTPs, 15 µL each 10 µM forward and reverse primers, 6 µL 10 nM oligonucleotide library, 3 µL Q5 DNA polymerase, and 186 µL H_2_O with the following thermocycling parameters: 98°C, 30 s; 15 cycles of the following: 98°C, 15 s; 56°C, 15 s; 72°C, 15 s; 72°C, 10 min; and 10°C, hold. Spin-purified PCR products were digested with BsaI-HF-v2 (R3733; NEB), and the size and integrity of full-length and digested PCR products were confirmed on a 4% agarose e-gel (Thermo). The BsaI-digested PCR product (without further purification) was ligated into a BsaI-digested MCi plasmid as detailed in reference 47. The ligation was purified by spot dialysis on a nitrocellulose filter (Millipore VSWP02500) against 0.1 mM Tris, pH 8 buffer for 20 min prior to transformation by electroporation into *E. coli* strain BW25141 (sJMP146). Cells were plated at a density of ~50,000 cells/plate on 150 mm LB-2% agar plates supplemented with carbenicillin. After incubation for 18 h at 37°C, colonies (~1,300,000 [Z1-genes], 700,000 [Z2-controls], and 1,950,000 [Z3-mismatches] for >30 coverage/oligonucleotide) were scraped from the agar plates into LB and pooled and the plasmid DNA was extracted from ~1 × 10^11^ cells (10 mL at OD_600_ = 33 [Z1], 25 mL at OD_600_ = 11 [Z2], and 10 mL at OD_600_ = 36 [Z3]) using a Midiprep Kit. This pooled Mobile-CRISPRi library was transformed by electroporation into *E. coli* mating strain sJMP3049 (20 ng plasmid DNA plus 90 µL electrocompetent cells, plated at a density of ~30,000 cells/plate on 150 mm LB-2% agar plates supplemented with carbenicillin and DAP). After incubation for 18 h at 37°C, colonies (~935,000 [Z1-genes], 124,000 [Z2-controls], and 247,000 [Z3-mismatches]) were scraped from the agar plates and pooled and resuspended in LB with DAP and 15% glycerol at OD_600_ (40 [Z1], 17 [Z2], and 34 [Z3]) and aliquots of the pooled CRISPRi libraries were stored as strains sJMP2618, 2619, and 2620 (Z1-genes, Z2-controls, and Z3-mismatches, respectively) at −80°C. Combined results from Z1-genes and Z2-controls are reported in this paper.

### Transfer of the Mobile-CRISPRi system to the *E. coli* or *Z. mobilis* chromosome

The MCi system was transferred to the Tn*7att* site on the chromosome of *Z. mobilis* by tri-parental conjugation of two donor strains—one with a mobilizable plasmid (pTn7C1) encoding Tn7 transposase and a second with a mobilizable plasmid containing a Tn7 transposon encoding the CRISPRi system—and the recipient strain *Z. mobilis* ZM4. All matings used the *E. coli* WM6026 donor strain, which is *pir^+^* to support pir-dependent plasmid replication, *dap*^−^, making it dependent on diaminopimelic acid for growth, and encodes the RP4 transfer machinery required for conjugation. A detailed mating protocol for strains with individual sgRNAs was described previously (29, 47). Briefly, *E. coli* strains were grown ~16 h and Z. *mobilis* strains were grown ~24 h from single colonies. Cultures were spun at 4,000 × *g* for 5 min, and the cell pellets were washed twice with and equal volume of media (no antibiotic or DAP). For *E. coli* recipients, 100 µL of the washed donor and recipient strains was added to 700 µL LB and pelleted at ~4,000 × *g* and the cells were placed on a nitrocellulose filter (Millipore HAWP02500) on an LB plate supplemented with DAP and incubated at 37°C, ~2 h. For *Z. mobilis* recipients, 100 µL of the washed culture of donor strains and 500 µL of the recipient strain were added to 300 µL RMG and pelleted at ~6,000 × *g* and the cells were placed on a nitrocellulose filter (Millipore HAWP02500) on an RMG plate supplemented with DAP and incubated at 30°C, ~24 h. Cells were removed from the filter by vortexing in 200 µL media, serially diluted, and grown with selection on LB-cm plates at 37°C (*E. coli*) or selection on RMG-cm plates at 30°C (*Z. mobilis*).

For pooled library construction, aliquots of the Tn*7* transposase donor strains (sJMP2618, 2619, and 2620) and pooled library strains were thawed and diluted to OD_600_ = 10 in RMG. Overnight cultures of the *E. coli* Tn*7* transposase donor (sJMP2591) and *Z. mobilis* recipient strain (sJMP412) were spun down for 10 min at 4,000 × *g*, and the OD_600_ was normalized to 10. For each library, 2 mL of each strain was mixed and centrifuged for 10 min at 6,000 × *g*. Pelleted cells were spotted on two RMG agar plates and incubated for 39 h at 30°C prior to resuspension in RMG, serial dilution, and plating ~40,000 CFU/150 mm RMG-cm plates solidified with 2% agar followed by incubation for 72 h at 30°C. Cells were scraped from plates and resuspended in RMG + 15% glycerol, the density was normalized to OD_600_ = 9, and aliquots were stored at −80°C as strains sJMP2621, 2622, and 2623. Efficiency of trans-conjugation (colony forming units on RMG-cm vs. RMG) was ~1 in 10^4^.

### Analysis of Mobile-CRISPRi reverse polarity in *Z. mobilis* or *E. coli* using a fluorescent reporter operon

An operon encoding mScarlet and sfGFP reporter genes was cloned into the Mobile-CRISPRi vector (pJMP2367) at the PmeI site. Nine sgRNAs per reporter gene were then designed and individually cloned into this plasmid vector, and the Mobile-CRISPRi constructs were transferred to *Z. mobilis* as described above.

For *Z. mobilis*, cultures from single colonies were grown in triplicate in 1 mL RMG-cm in a 96-well deep well plate covered with AeraSeal at 30°C without shaking for ~24 h. *Z. mobilis* was subcultured 1:1,000 inoculum:medium into two 96-well deep well plates containing either RMG or RMG + 1 mM IPTG and incubated as described above. The plates were centrifuged at 4,000 × *g* for 10 min, the supernatant was removed, and the resulting cell pellets were resuspended in 1 mL PBS. Next, 200 µL of the cell suspension was transferred to a clear-bottom black microplate and measured in a Tecan Infinite microplate reader. Cells were shaken for 30 s (linear amplitude: 2.5 mm) prior to measurement of cell density (OD_600_), GFP fluorescence (482/515 nm excitation/emission), and mScarlet fluorescence (560/605 nm). Experiments were repeated three times with 2–4 biological replicates each.

*E. coli* experiments were performed similarly, with the following modifications: LB was used in place of RMG, overnight growth was in 300 µL with shaking at 37°C for ~18 h, *E. coli* was subcultured 1:10,000 inoculum:medium and grown 6–7 h, and *E. coli* cells were further diluted 1:2 in PBS (to OD_600_ ~ 0.3–0.6) prior to measurement to avoid cell shadowing in dense culture.

OD-corrected fluorescence is reported relative to a no-sgRNA fluorescent control. One sgRNA (“S2,” which targets mScarlet) proved toxic to *Z. mobilis* and thus was excluded from analysis for this organism.

### Generation of predicted transcription units from RNA-seq data

We used previously published RNA sequencing (RNA-seq) data (GSE139939) gathered from *Z. mobilis* ZM4 cultures grown to exponential and stationary phase in RMG under aerobic and anaerobic conditions to generate putative transcription unit assignments for *Z. mobilis* (11). Briefly, after adapter trimming with Cutadapt version 2.10 (91) and quality trimming with Trimmomatic version 0.39 (92), we aligned replicates from each condition and growth phase separately to the ZM4 reference chromosome (GenBank CP023715.1) and large plasmids (GenBanks CP023716.1, CP023717.1, CP023718.1, and CP023719.1) using Rockhopper version 2.03 (93–95) to generate a set of predicted TUs for each condition and growth phase. Using custom Python scripts and Scipy version 1.5.2 (96), predicted transcript isoforms that covered the same genes for each growth phase were then parsed from Rockhopper output files and merged through single linkage hierarchical clustering using a pseudo-distance metric between two transcripts of “max size of compared transcripts in basepairs - number of basepairs that overlap” and a cophenetic cutoff of 100 bp. For merged transcripts, the most extreme boundaries were used. TU assignments are included in Table S5.

### Library growth experiment

*Z. mobilis* CRISPRi libraries were incubated either in air or in an anaerobic chamber. The *Z. mobilis* CRISPRi libraries (sJMP2621, 2622, and 2623) were revived by the addition of 100 µL total frozen library stocks (OD_600_ = 9, 8:1:6 ratio Z1:Z2:Z3) into 100 mL RMG (starting OD_600_ = ~0.01) in a 500-mL flask and incubated with stirring at 150 rpm at 30°C until OD_600_ = ~0.2–0.3 (~8 h) (initial timepoint = *T*_i_) in duplicate. These cultures were diluted back to OD_600_ = 0.01 in RMG + 1 mM IPTG in duplicate and incubated until OD_600_ = ~0.2–0.3 (~8 h) (final timepoint = *T*_f_), at which point they were again diluted back to OD_600_ = 0.01 in RMG + 1 mM IPTG in duplicate and incubated until OD_600_ = ~0.2–0.3 (~8 h). Cells were pelleted at 6,000 × *g* from 30 mL (*T*_i_) or 10 mL (*T*_f_), washed with 1× PBS and stored at −20°C for DNA extraction. Aerobic and anaerobic experiments were each done twice on separate days.

### Sequencing library samples

DNA was extracted from cell pellets with the DNeasy gDNA Extraction Kit (Qiagen) according to the manufacturer’s protocol, resuspending in a final volume of 100 µL with an average yield of ~50 ng/µL. The sgRNA-encoding region was amplified using Q5 DNA polymerase (NEB) in a 100-µL reaction with 2 µL gDNA (~100 ng) and primers oJMP697 and oJMP698 (nested primers with partial adapters for index PCR with Illumina TruSeq adapter) according to the manufacturer’s protocol using a Bio-Rad C1000 thermal cycler with the following program: 98°C, 30 s, and then 16 cycles of the following: 98°C, 15 s; 65°C, 15 s; and 72°C, 15 s. PCR products were spin purified and eluted in a final volume of 20 µL for a final concentration of ~20 ng/µL. Samples were sequenced by the UW-Madison Biotech Center Next Generation Sequencing Core facility. Briefly, PCR products were amplified with nested primers containing i5 and i7 indexes and Illumina TruSeq adapters followed by bead cleanup, quantification, pooling, and running on a NovaSeq 6000 (150 bp paired-end reads).

### Counting sgRNA sequences

sgRNA-encoding spacer sequences were counted using the seal.sh script from the BBTools package (release: 28 March 2018). Briefly, paired FASTQ files from amplicon sequencing were aligned in parallel to a reference file that contained the spacer sequences cloned into the library. Alignment was performed using *k*-mers of 20 nucleotides in length—equal to the length of the spacer sequence. For more information on digital resources and links to custom scripts, see Table S3.

### Comparisons between conditions

Log_2_ fold change and confidence intervals were computed using edgeR (97). Briefly, trended dispersion of sgRNA-encoding spacers was estimated and imputed into a quasi-likelihood negative binomial log-linear model. Changes in abundance and the corresponding false discovery rates were computed for each spacer in each condition. Finally, gene-level fitness scores were obtained by calculating the median LFC of the spacers targeting each gene; gene-level significance was calculated by computing the Stouffer’s *P*-value (poolr R package) using the FDRs of the spacers targeting each gene (Table S8). For more information on digital resources and links to custom scripts, see Table S3.

### Classifying gene essentiality and fitness defects

Genes with significance ≤ 0.05 and median LFC ≤ −3 under aerobic and anaerobic conditions were considered generally essential. Genes with significance ≤ 0.05 and median LFC ≤ −3 under only one condition were classified as conditionally essential. Genes with significance ≤ 0.05 and median LFC ≤ −1 in one or both conditions were classified as having a fitness defect in the respective condition(s).

### Analysis of individual CRISPRi knockdown strains for essentiality using spot dilution assays

Phenotypes of gene knockdowns identified in the pooled screen were assayed by constructing individual *Z. mobilis* strains with sgRNAs targeting 31 putative generally essential genes, 20 putative aerobic essential or aerobic-specific fitness defect genes, 24 putative anaerobic essential or anaerobic-specific fitness defect genes, and 6 non-targeting control sgRNA strains. Guides used for follow-up experiments were distinct from the ones found in our pooled screen but were designed using the same parameters. Individual colonies from each strain were picked in duplicate (two biological replicates) into 1 mL RMG-cm into 96-well deep well plates, covered with AeraSeal, and incubated at 30°C for 24 h to saturation without shaking. Cultures were serially (1:10) diluted in RMG in a sterile V-bottom 96-well microplate and spotted onto RMG or RMG + 1 mM IPTG agar plates (OmniTray, Thermo 242811, 33 mL 2% agar medium) using a 96-well manual pinning tool (V&P Scientific; VP407A pin tool, VP381N microplate aligner, and VP380 OmniTray agar plate aligner) in triplicate (three technical replicates) and incubated either aerobically or anaerobically at 30°C for 72 h. Growth was measured using a fitness score and colony size score. Fitness scores represent the number of serial dilutions resulting in a visible spot, relative to a non-targeting control (i.e., a knockdown mutant with a fitness defect that produced spots for five dilutions, in comparison to a non-targeting control strain that produced spots for six dilutions, would have a fitness score equal to −1). Colony sizes were scored as small, large, or control-like. Growth was scored by three individuals; the median fitness score and the most prevalent colony size score are reported.

### Analysis of *recJ* CRISPRi knockdown strain mutation rate in the presence of rifampicin

Acquisition of rifampicin resistance by a *Z. mobilis recJ* knockdown strain (sJMP10367) compared with a non-targeting control strain (sJMP10366) and parent strain (sJMP10365) was assayed as follows: ~2.4 × 10^6^ cells resuspended off plates into RMG (200 µL of a 10^−5^ dilution of cells normalized to OD_600_ = 9) were plated in triplicate on RMG + 20 µM IPTG and incubated at 30°C for 90 h to induce partial knockdown. ~2.6 × 10^8^ cells from each replicate resuspended off plates into RMG (200 µL of cells normalized to OD_600_ = 12) were plated in quadruplicate on RMG or RMG + 4 µM rifampicin (rif) and incubated aerobically and on RMG or RMG + 8 µM rif and incubated anaerobically, at 30°C in the dark for 96 h. Colony forming units were compared for growth ± rif.

### Fitness measurement of *Z. mobilis atp* and *rnf* partial knockdowns with ion-motive force disruption by CCCP

Fitness challenge experiments were performed in clear 96-well microplates inoculated 1:50 (inoculum:medium) in duplicate from a saturated 5-mL overnight culture grown in RMG under either aerobic or anaerobic conditions. For anaerobic experiments, the 96-well microplate was placed in the anaerobic chamber overnight prior to inoculation. *atp* knockdown (sJMP6104, sJMP6105, and sJMP6109), *rnf* knockdown (sJMP6103), and non-targeting CRISPRi (sJMP6101) strains were grown in RMG with sublethal concentrations of inducer (50 µM IPTG for Fig. 4; Fig. S5; 25–50 μM IPTG for Fig. S4), carbonyl cyanide 3-chlorophenylhydrazone (8 µg/mL, from stock dissolved in DMSO), and/or DMSO (0.5%) as a control. Growth data were collected in a Tecan Sunrise plate reader for ~48 h. Experiments were repeated three times with two biological replicates each.

Actual relative fitness was measured by calculating the empirical area under the curve (auc_e) for each growth curve using the Growthcurver R package (version 0.3.1) (98) relative to the DMSO-only control for each strain. Following the multiplicative theory for synergy (49), predicted relative fitness for a combination of two conditions was calculated by multiplying the actual relative fitness values obtained from each condition individually (i.e*.,* induction and CCCP). Actual and predicted fitness values are reported relative to those of the non-targeting control strain.

### Detection of membrane potential using Thioflavin T fluorescence

Overnight cultures of *rnfE* knockdown (MT271), *atpC* knockdown (MT272), and a non-targeting CRISPRi strain (MT270) were inoculated from single colonies in ZRDM + 70 µg/mL cm in duplicate and grown at 30°C in an anaerobic chamber. Cultures were diluted in fresh ZRDM-cm + 10 µM ThT to OD_600_ = 0.04. Fifty micromolar-IPTG inducer was added as indicated. Two hundred microliters from each biological replicate was transferred to a 96-well microplate (flat, black, clear bottom, Greiner) in triplicate. Cells were grown at 30°C with shaking in a BioTek Synergy HTX Multimode Reader in an anaerobic chamber. OD_600_ and ThT fluorescence (optical filters 460/40 nm and 528/20 nm for excitation and emission, respectively) were measured every 15 min for 30 h. Average ThT fluorescence and OD_600_ values were corrected by subtracting background values from abiotic controls along the time course.

### *Z. mobilis* growth curves in RMG or ZRDM

Aerobic cultures grown overnight in 5 mL RMG were washed twice in ZMM and then resuspended in ZMM to original volume. The washed cultures were diluted 1:50 (2 µL into 98 µL) into growth medium (RMG or ZRDM) in a 96-well microplate and grown ~24 h in a Tecan Sunrise microplate reader in the anaerobic chamber. Growth curves represent four to six biological replicates across two to three experiments (Fig. S8).

### Liquid chromatography-mass spectrometry metabolomics for *atp* and *rnf* knockdown strains

*Z. mobilis* was grown at 30°C in an anaerobic chamber. *Z. mobilis* cultures of *atp* knockdowns (sJMP6104, sJMP6105, and sJMP6109), *rnfE* knockdown (sJMP6103), and non-targeting CRISPRi (sJMP6101) strains were grown overnight, stationary, in 10 mL RMG in test tubes from single colonies, diluted ~1:1,000 into 60 mL RMG in 125 mL flasks, and grown with stirring (120 rpm) until OD_600_ = ~0.7–0.9 to use as inoculum for experimental flasks. Experimental flasks were inoculated to OD_600_ = 0.05 in 100 mL ZRDM + 50 µM IPTG in 250-mL flasks with stirring. From this flask, 30 mL was transferred into each of three separate 125-mL flasks with stirring. When these cultures reached OD = 0.5, 10 mL from each experimental flask was harvested by vacuum filtration onto a 0.45-µm nylon membrane filter (47 mm in diameter). Cells were resuspended off the filter into 1.5-mL ice cold solvent (40:40:20 acetonitrile:methanol:water), and the solvent was stored in a microcentrifuge tube at −80°C until ready for same-day processing.

As an internal standard, wild-type *Z. mobilis* ZM4 was grown in ZMM + labeled glucose (Table S1). Wild-type *Z. mobilis* was prepared as described above, with the following modifications: overnight RMG cultures were diluted into 10 mL ZMM + labeled glucose, a single experimental flask containing 30 mL ZMM + labeled glucose was inoculated, and 10 mL was collected in duplicate from the experimental flask.

Collected samples were spun in a microcentrifuge at max speed for 10 min at 4°C. Each sample’s supernatant was mixed 1:1 with supernatant from the labeled glucose internal standard and dried under a nitrogen gas stream.

Metabolomics LC-MS analyses (Table S9) were conducted utilizing a Vanquish ultra-high-performance liquid chromatography (UHPLC) system (Thermo Scientific), coupled with a Q Exactive hybrid quadrupole-Orbitrap mass spectrometer (Thermo Scientific) using electrospray ionization in negative-ion mode. The chromatography was performed at 25°C with a reverse-phase C18 column of 2.1 × 100 mm with 1.7 µm particle size (Water from Acquity UHPLC BEH). Solvent A (97:3 H_2_O:methanol with 10 mM tributylamine adjusted to pH 8.2 using 10 mM acetic acid) and Solvent B (100% methanol) were used in a gradient manner: 0–2.5 min with 5% B, 2.5–17 min with a linear gradient from 5% B to 95% B, 17–19.5 min with 95% B, 19.5–20 min with a linear gradient from 95% B to 5% B, and 20–25 min with 5% B. The flow rate was constant at 0.2 mL/min. For targeted metabolomics, the eluent was injected into the MS for analysis until 18 min, after which the flow was redirected to waste. The MS parameters included full MS-SIM (single ion monitoring) scanning between 70 and 1,000 m/z and 160 and 815 m/z for the targeted metabolomics and MEP metabolite-specific methods, respectively. The automatic control gain (ACG) target was 1e6, with a maximum injection time (IT) of 40 ms and a resolution of 70,000 full width at half maximum (FWHM).

## Data Availability

CRISPRi amplicon sequencing reads have been deposited to the National Center for Biotechnology Information Sequencing Read Archive (SRA) under BioProject PRJNA1018248.

## SUPPLEMENTAL MATERIAL

Supplemental Figures mbio.01487-23-s0001.pdf Fig. S1-S8.

Table S1 mbio.01487-23-s0002.xlsx Strains, plasmids, oligos, and media.

Table S2 mbio.01487-23-s0003.xlsx Ortholog conservation.

Table S3 mbio.01487-23-s0004.xlsx Digital resources.

Table S4 mbio.01487-23-s0005.xlsx Library key.

Table S5 mbio.01487-23-s0006.xlsx Transcription unit predictions.

Table S6 mbio.01487-23-s0007.xlsx Essential genes.

Table S7 mbio.01487-23-s0008.xlsx Uncharacterized and unique genes with phenotypes.

Table S8 mbio.01487-23-s0009.xlsx All gene and spacer log2 fold changes.

Table S9 mbio.01487-23-s0010.xlsx Metabolomics data.

ASM does not own the copyrights to Supplemental Material that may be linked to, or accessed through, an article. The authors have granted ASM a non-exclusive, world-wide license to publish the Supplemental Material files. Please contact the corresponding author directly for reuse.
