# Supplementary material for: The genetics of aerotolerant growth in an alphaproteobacterium with a naturally reduced genome
Source: mBio. 2023 Oct 31;14(6):e01487-23. doi: 10.1128/mbio.01487-23 (PMC10746277; doi:10.1128/mbio.01487-23)
Supplement: Supplemental Figures — Fig. S1-S8. [file mbio.01487-23-s0001.pdf]

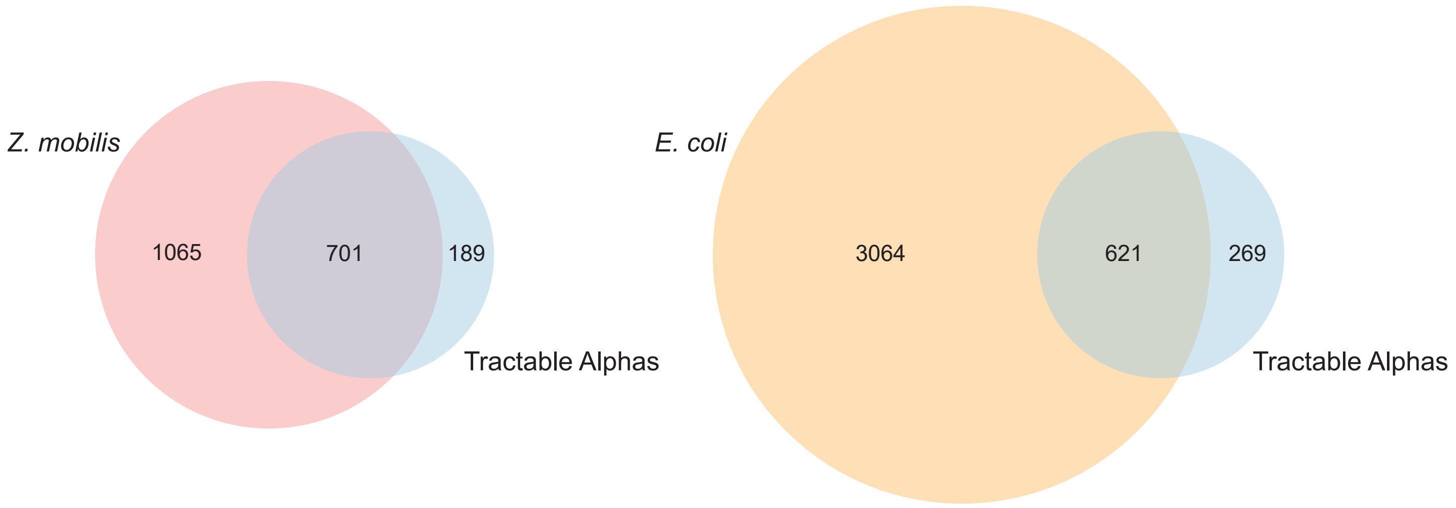

Figure S1. Gene homology of *Z. mobilis* or *E. coli* with other Alphaproteobacteria. Blue overlapping circles represent protein-coding orthologs shared across all seven Tractable Alphas (*C. crescentus, B. subvibrioides, A. tumefaciens, B. diazoefficiens, R. palustris, R. sphaeroides, S. wittichii*). Red and orange overlapping circles represent all protein-coding orthologs in *Z. mobilis* and *E. coli*, respectively.

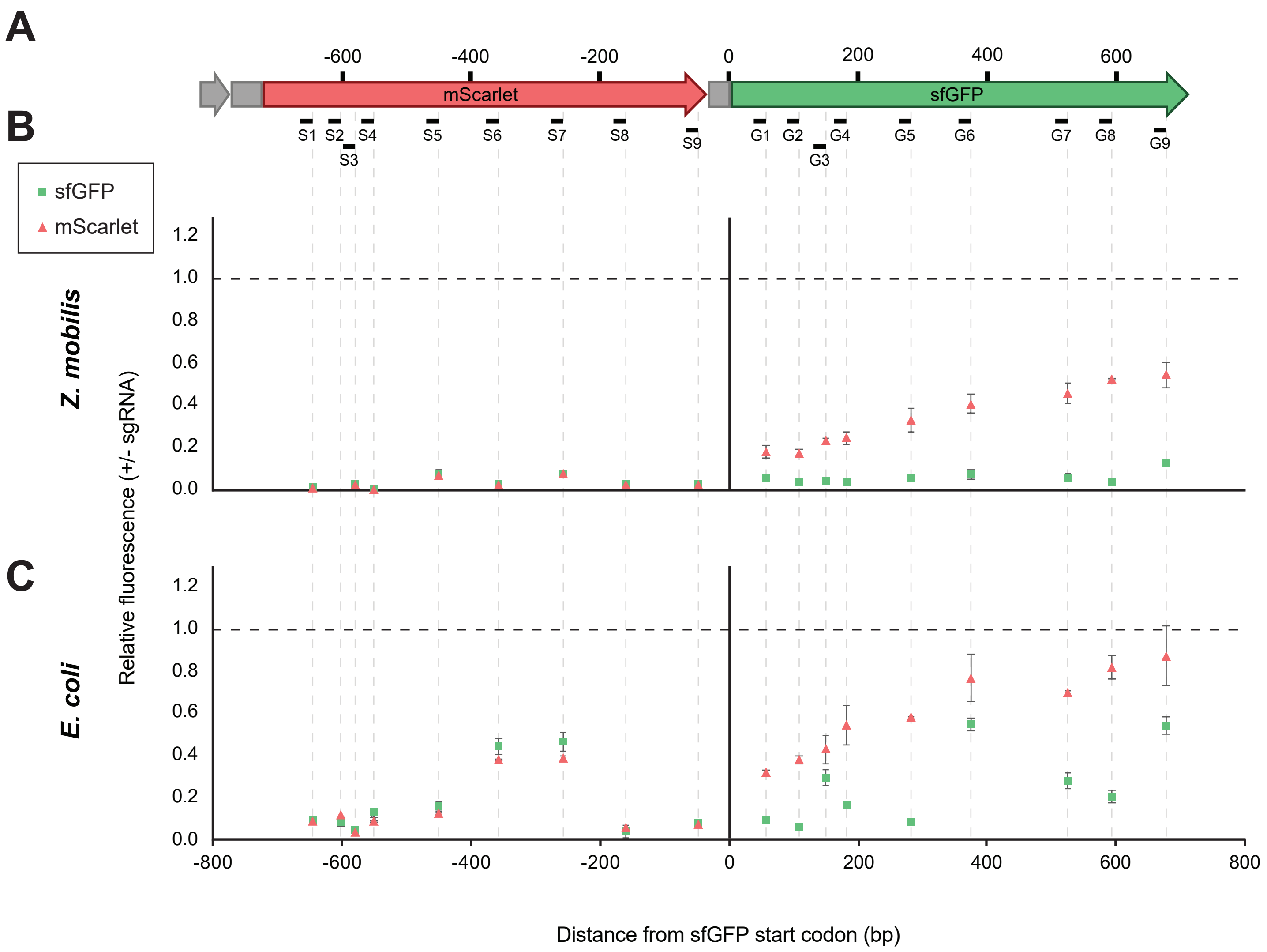

Figure S2. Reverse polarity in *Z. mobilis* CRISPRi. (A) Fluorescent reporter operon structure. sgRNAs targeting *mScarlet* (S1-9) and *sfGFP* (G1-9) are noted. (B-C) Fluorescence of mScarlet (pink triangle) and sfGFP (green square) for corresponding knockdown mutants in (B) *Z. mobilis* and (C) *E. coli*. Fluorescence is reported relative to a non-targeting sgRNA control (horizontal dashed line). Data represent three experiments with 2-4 biological replicates each. Error bars show standard deviation.

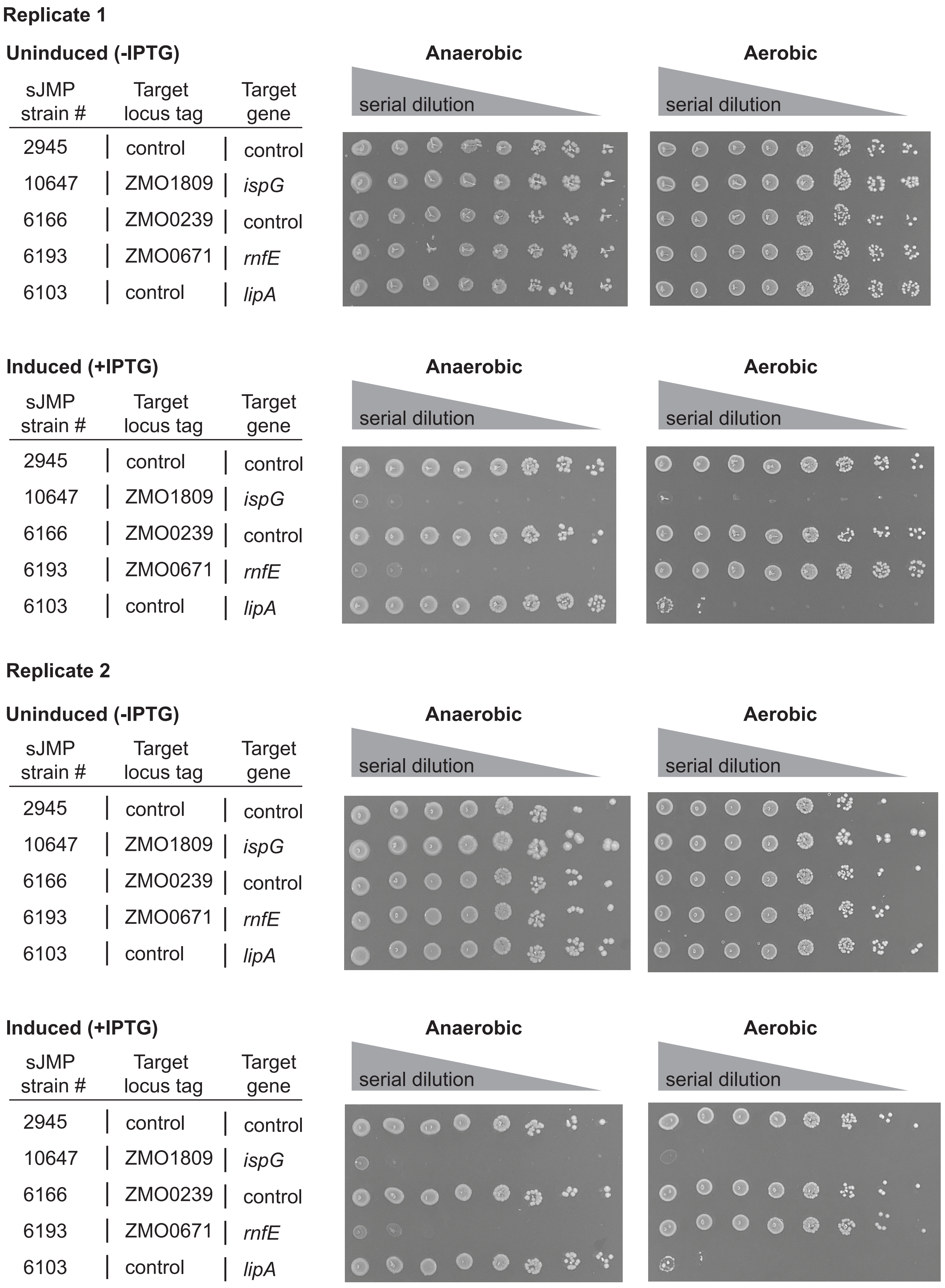

Figure S3. Example verification of *Z. mobilis* CRISPRi library screen phenotypes for generally essential (*ispG*), anaerobic essential (*rnfE*), and aerobic essential (*lipA*) genes using spot plates (see Methods).

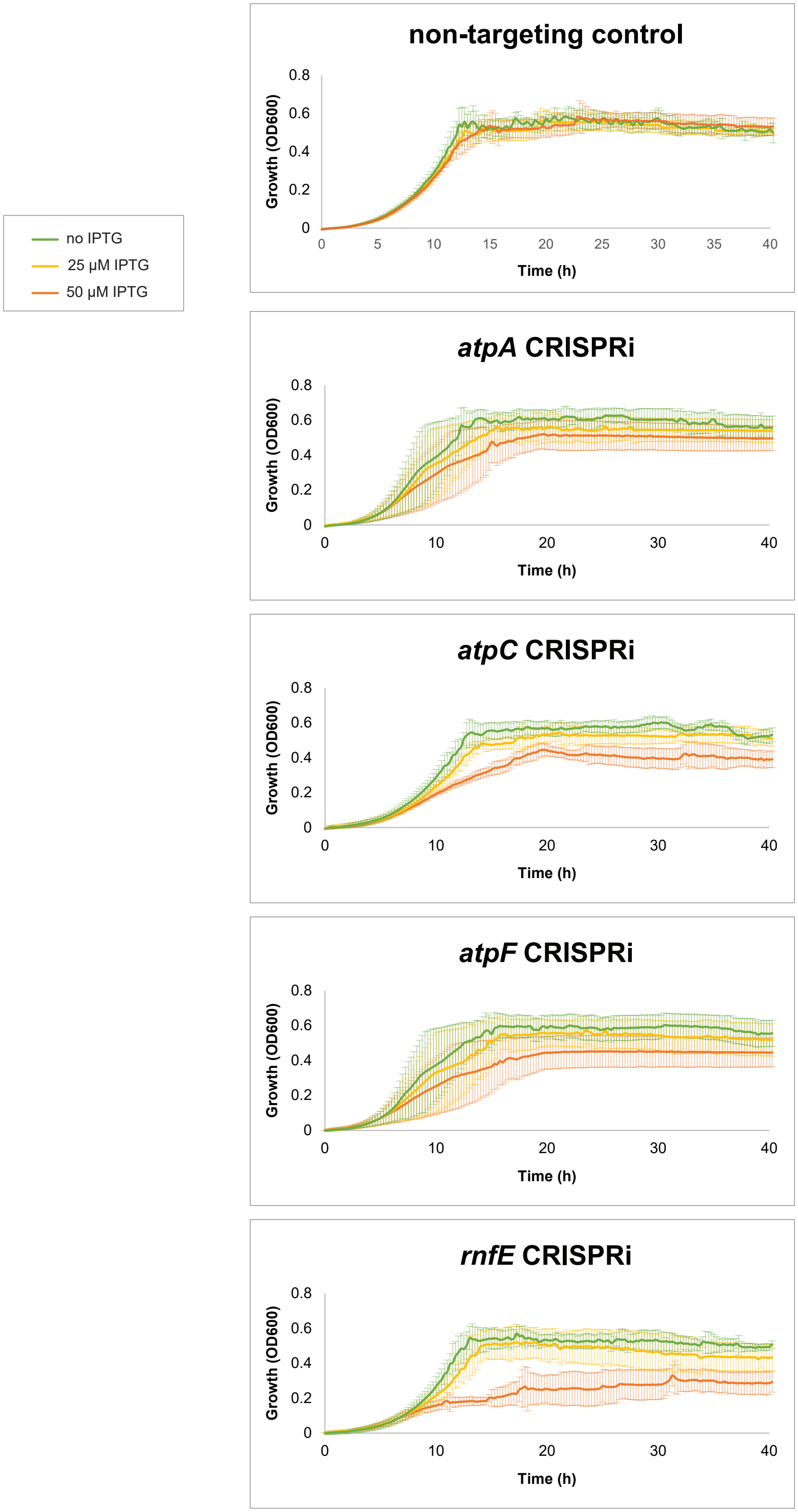

Figure S4. Anaerobic growth curves of CRISPRi strains from Fig. 4, with varying levels of IPTG to induce partial knockdown. Green, no IPTG; yellow, 25 μM IPTG; orange, 50 μM IPTG. Data represent six replicates across three experiments. Error bars show standard deviation. DMSO (0.5%) was used for all growth curves to control for DMSO solvent for CCCP in Fig. 4.

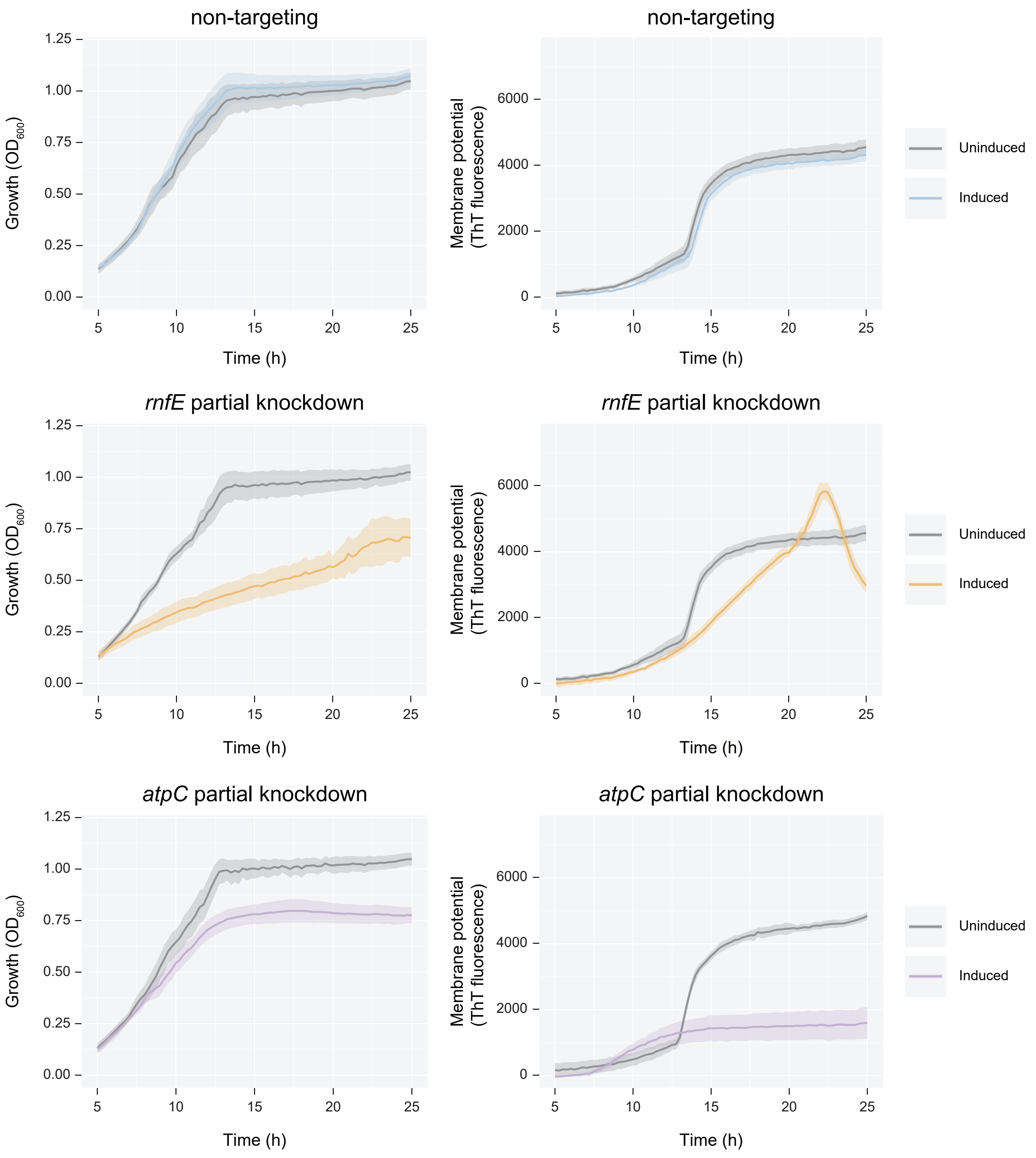

Figure S5. Growth (left column) and membrane potential (right column) for strains corresponding to Figure 4B. Shaded ribbons represent one standard deviation from the mean. Data represent six replicates.

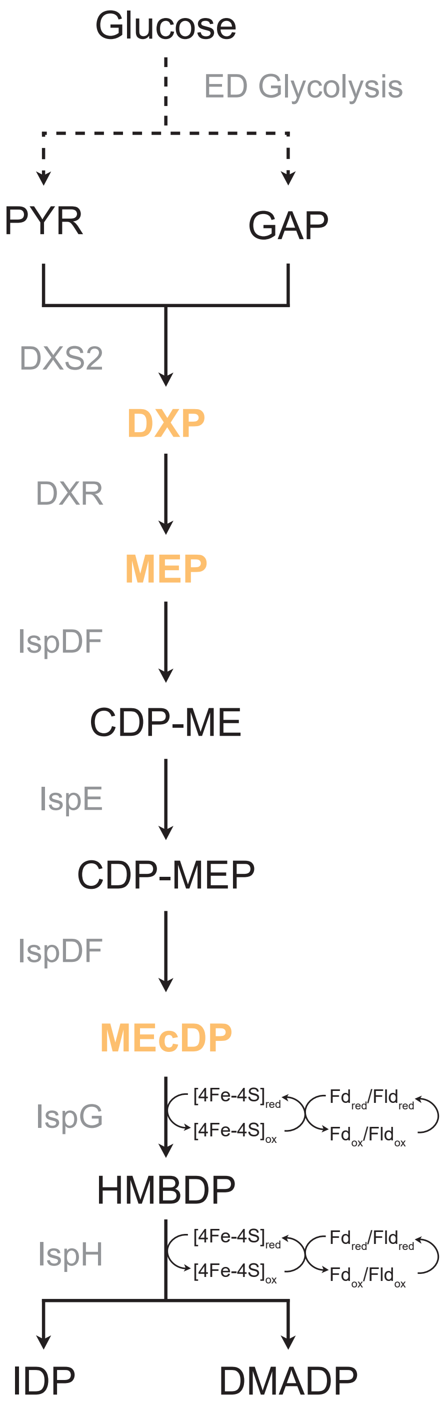

Figure S6. *Z. mobilis* isoprenoid biosynthesis through the MEP pathway. Relative abundances of intermediates in orange text are reported in this study. ED, Entner-Doudoroff (ED); PYR, pyruvate; GAP, glyceraldehyde 3-phosphate; DXP, 1-deoxy-d-xylulose 5-phosphate; MEP, 2-C-methyl-d-erythritol 4-phosphate; CDP-ME, 4-diphosphocytidyl-2-C-methyl-d-erythritol; CDP-MEP, 4-diphosphocytidyl-2-C-methyl-d-erythritol 2-phosphate; MEcDP, 2-C-methyl-d-erythritol 2,4-cyclodiphosphate; HMBDP, 4-hydroxy-3-methylbut-2-enyl-diphosphate; IDP, isopentenyl diphosphate; DMADP, dimethylallyl diphosphate. DXS2, DXP synthase; DXR, DXP reductoisomerase; IspDF, MEP cytidyl transferase/MEcDP synthase; IspE, CDP-ME kinase; IspG, HMBDP synthase; IspH, HMBDP reductase; Fd, ferredoxin; Fld, flavodoxin. Figure adapted from Khana and Tatli, *et al*. (82).

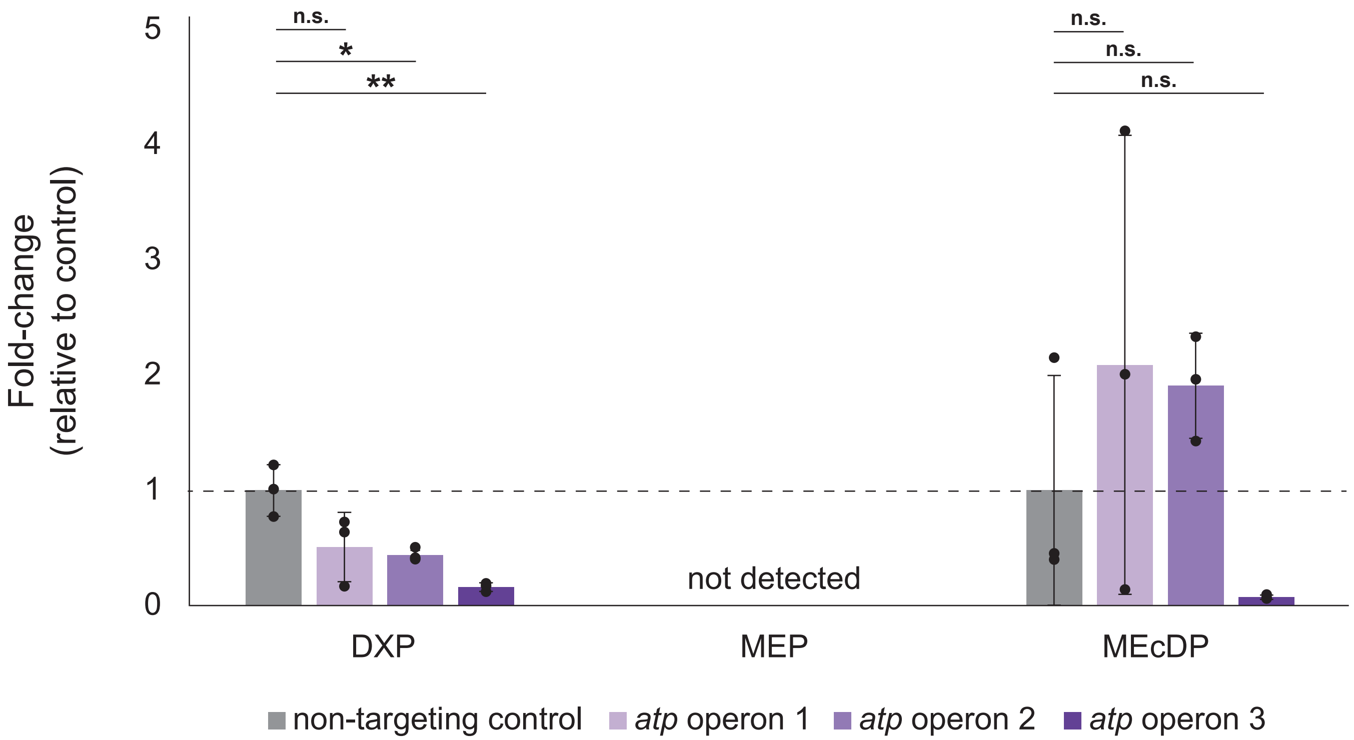

Figure S7. Mass spectroscopy metabolomics measurement of MEP pathway intermediates for (leftmost gray bars and dashed line) non-targeting CRISPRi control and (righthand purple bars) *atp* partial knockdown. n.s., not significant; *, $p < 0.05$; **, $p < 0.01$ by two-tailed Student's T-test. Data represent three replicates. Error bars show standard deviation. *atp* operon 1 knockdown targets *atpA*; *atp* operon 2 knockdown targets *atpC*; *atp* operon 3 knockdown targets *atpF*. DXP, 1-deoxy-D-xylulose 5-phosphate; MEP, 2-C-methyl-D-erythritol 4-phosphate; MEcDP, 2-C-methyl-D-erythritol-2,4-cyclodiphosphate.

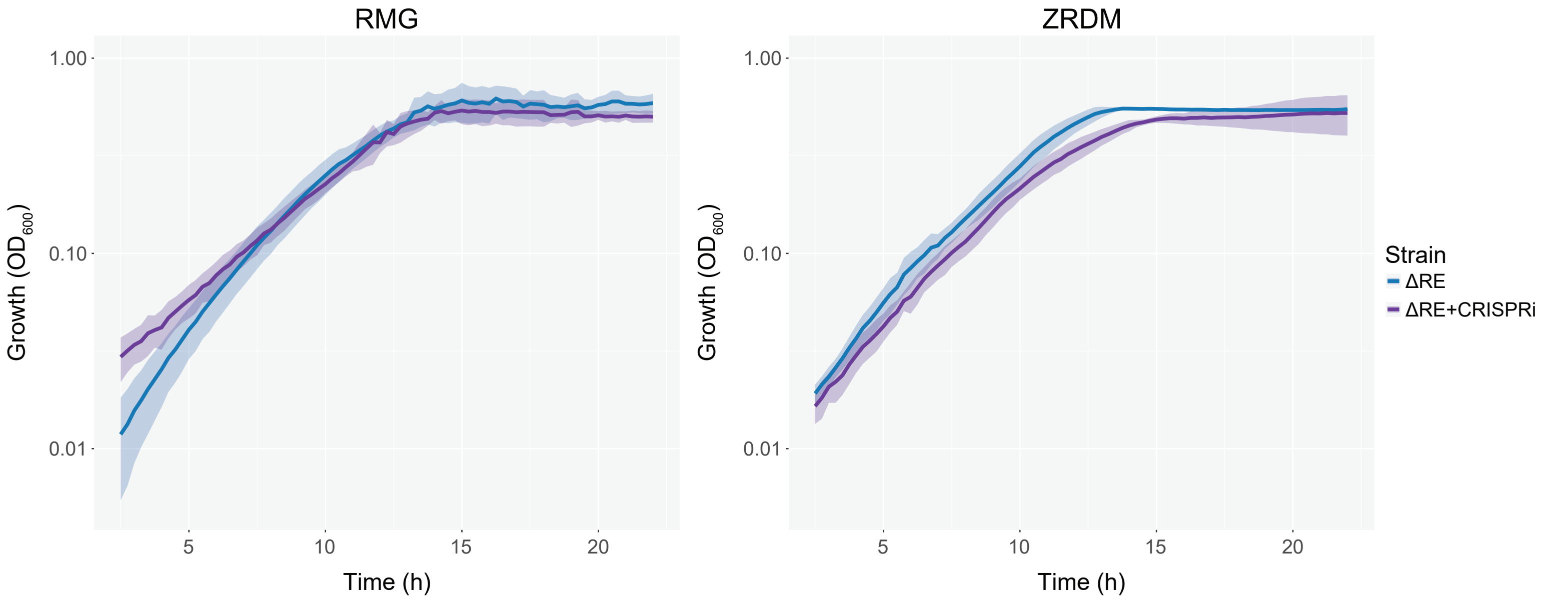

Figure S8. Growth of *Z. mobilis* strains in Rich Medium with Glucose (RMG) or *Zymomonas* Rich Defined Medium (ZRDM) without induction. ΔRE, restriction-deficient parent strain (sJMP412); ΔRE+CRISPRi, ΔRE strain with non-targeting Mobile-CRISPRi (sJMP2554). Shaded ribbons represent one standard deviation from the mean. Data represent 4-6 replicates across 2-3 experiments.
